# ZP4 Is Present in Murine Zona Pellucida and Is Not Responsible for the Specific Gamete Interaction

**DOI:** 10.3389/fcell.2020.626679

**Published:** 2021-01-18

**Authors:** Mª José Izquierdo-Rico, Carla Moros-Nicolás, Míriam Pérez-Crespo, Ricardo Laguna-Barraza, Alfonso Gutiérrez-Adán, Frédéric Veyrunes, José Ballesta, Vincent Laudet, Pascale Chevret, Manuel Avilés

**Affiliations:** ^1^Department of Cell Biology and Histology, Faculty of Medicine, University of Murcia, Murcia, Spain; ^2^Institute for Biomedical Research of Murcia (IMIB-Arrixaca), Murcia, Spain; ^3^International Excellence Campus for Higher Education and Research “Campus Mare Nostrum”, Murcia, Spain; ^4^Department of Animal Reproduction, Instituto Nacional de Investigacion y Tecnologia Agraria y Alimentaria (INIA), Madrid, Spain; ^5^Institut des Sciences de l'Evolution, UMR5554 CNRS/Université Montpellier/IRD/EPHE, Montpellier, France; ^6^Marine Eco-Evo-Devo Unit, Okinawa Institute of Science and Technology, Okinawa, Japan; ^7^Laboratoire de Biométrie et Biologie Evolutive, UMR5558, CNRS, Université de Lyon, Université Claude Bernard Lyon 1, Villeurbanne, France

**Keywords:** zona pellucida, ZP4, *Mus musculus*, murine phylogeny, oocyte, pseudogene, sperm

## Abstract

Mammalian eggs are surrounded by an extracellular matrix called the zona pellucida (ZP). This envelope participates in processes such as acrosome reaction induction, sperm binding, protection of the oviductal embryo, and may be involved in speciation. In eutherian mammals, this coat is formed of three or four glycoproteins (ZP1–ZP4). While *Mus musculus* has been used as a model to study the ZP for more than 35 years, surprisingly, it is the only eutherian species in which the ZP is formed of three glycoproteins Zp1, Zp2, and Zp3, *Zp4* being a pseudogene. *Zp4* was lost in the *Mus* lineage after it diverged from *Rattus*, although it is not known when precisely this loss occurred. In this work, the status of Zp4 in several murine rodents was tested by phylogenetic, molecular, and proteomic analyses. Additionally, assays of cross *in vitro* fertilization between three and four ZP rodents were performed to test the effect of the presence of Zp4 in murine ZP and its possible involvement in reproductive isolation. Our results showed that *Zp4* pseudogenization is restricted to the subgenus *Mus*, which diverged around 6 MYA. Heterologous *in vitro* fertilization assays demonstrate that a ZP formed of four glycoproteins is not a barrier for the spermatozoa of species with a ZP formed of three glycoproteins. This study identifies the existence of several mouse species with four ZPs that can be considered suitable for use as an experimental animal model to understand the structural and functional roles of the four ZP proteins in other species, including human.

## Introduction

The zona pellucida (ZP) is an extracellular coat that surrounds mammalian oocytes and early embryos. This envelope participates in important events during fertilization and early embryo development, such as the species-specific gamete recognition, acrosome reaction induction, preventing polyspermy, and protecting the oviductal embryo (Yanagimachi, 1994; Dean, 2007; Wassarman and Litscher, 2009; Gupta and Bhandari, 2011; Gupta et al., 2012; Tanihara et al., 2013; Shu et al., 2015). The composition of the ZP matrix has been elucidated in many species, and has been seen to be composed of three to four glycoproteins in eutherians (Bleil and Wassarman, 1980; Hedrick and Wardrip, 1987; Lefièvre et al., 2004; Hoodbhoy et al., 2005; Ganguly et al., 2008; Goudet et al., 2008; Izquierdo-Rico et al., 2009; Stetson et al., 2012, 2015; Moros-Nicolás et al., 2018c), and four to seven in marsupials and monotremes (Frankenberg and Renfree, 2018; Moros-Nicolás et al., 2018a; Wu et al., 2018). Indeed, the composition of ZP is more variable than was previously expected. Eutherian mammals can be classified into three categories according to their ZP protein composition: (a) species with four glycoproteins (ZP1, ZP2, ZP3, and ZP4) such as human, rat, hamster, horse, rabbit, cat, cheetah, ferret, tiger, panda, polar bear, and walrus (Lefièvre et al., 2004; Hoodbhoy et al., 2005; Izquierdo-Rico et al., 2009; Mugnier et al., 2009; Stetson et al., 2012, 2015; Moros-Nicolás et al., 2018c); (b) species whose ZP is formed of ZP2, ZP3, and ZP4 (pig, cow, marmoset, tarsier, dog, Weddell seal, and Antarctic fur seal) (Hedrick and Wardrip, 1987; Noguchi et al., 1994; Goudet et al., 2008; Stetson et al., 2012; Moros-Nicolás et al., 2018c); and (c) species whose ZP is formed of ZP1, ZP2, and ZP3 (house mouse) (Bleil and Wassarman, 1980).

Studies on the molecular evolution of the ZP family has helped to better understand the species-specific differences in the ZP composition. However, there is no consensus in relation with the ZP nomenclature or the number of ZP subfamilies (Spargo and Hope, 2003; Goudet et al., 2008; Feng et al., 2018; Wu et al., 2018). The first events in the ZP evolution occurred before the evolution of the first amphibians (Spargo and Hope, 2003). The ancestral *ZPC* gene and the precursor of *ZP2, ZP4, ZPD*, and *ZPAX* subfamilies appeared after a gene duplication event (Spargo and Hope, 2003). This precursor duplicated several times over a short period of evolutionary history, and led to the ancestral *ZPAX* gene and the ancestral of *ZP2, ZP4*, and *ZPD* genes. Afterwards, duplication events have occurred in several lineages, the most important during early evolution of the amniotes and giving rise to *ZP1* and *ZP4* groups within the *ZPB* subfamily (Hughes and Barratt, 1999; Bausek et al., 2000; Goudet et al., 2008). Thus, it was assumed that *ZP1* and *ZP4*, previously considered orthologs, are in fact paralogues. Some species retain the two copies of the ancestral gene (*ZP1* and *ZP4*, in the four glycoprotein model), and others conserved only one (*ZP1* or *ZP4*, the three glycoprotein model). In this last case, one of the copies (*ZP1* or *ZP4*) was lost after a duplication event due to a pseudogenization process (Goudet et al., 2008).

Massive gene loss events occurred during mammalian evolution (Goudet et al., 2008; Feng et al., 2018; Killingbeck and Swanson, 2018). For instance, there are several examples of *ZP1* loss in mammals; for example, in carnivores, a first pseudogenization event dated around 60–65 million years ago (MYA) (Nyakatura and Bininda-Emonds, 2012; Zhang et al., 2013) in the suborder caniformia [e.g., dog (*Canis familiaris*) (Goudet et al., 2008; Moros-Nicolás et al., 2018c) and fox (*Vulpes vulpes*) (Moros-Nicolás et al., 2018c)], and a second event after the separation of the Otariidae and Phocidae families, [e.g., Antarctic fur seal (*Arctocephalus gazella*) and Weddell seal (*Leptonychotes weddellii*) (Moros-Nicolás et al., 2018c)], estimated to have occurred around 22 MYA (Nyakatura and Bininda-Emonds, 2012). Another pseudogenization of *ZP1* took place early in the evolution of the Cetartiodactyla between 75 and 65 MYA (Zurano et al., 2019) as it was lost in the cow (*Bos taurus*), the dolphin (*Tursiops*) and the pig (*Sus crofa*) (Goudet et al., 2008; Stetson et al., 2012). *ZP1* was also probably independently lost twice in primates: in marmoset (*Callithrix*) and in tarsier (*Tarsius*) lineages (Stetson et al., 2012).

On the other hand, surprisingly, the pseudogenization of *ZP4* has been described only in the house mouse (*Mus musculus*) and in two South American marsupials (common opossum and gray short-tailed opossum) (Goudet et al., 2008; Moros-Nicolás et al., 2018a). In marsupials, this pseudogenization occurred after the split between the South American and Australasian marsupials dated at 80 MYA and before the divergence of common opossum and gray short-tailed opossum, between 20 and 30 MYA (Meredith et al., 2008; Jansa et al., 2014).

To date, ZP2 and ZP3 proteins are present in all species, which means that the functions of these proteins are essential; indeed, mouse Zp2 and Zp3 are indispensable for fertilization and embryo development (Liu et al., 1996; Rankin et al., 1996, 2001). Moreover, ZP2 was proven to be the primary sperm receptor in mice and human (Baibakov et al., 2012; Burkart et al., 2012; Avella et al., 2014, 2016).

Mouse ZP is formed of three proteins: Zp1, Zp2, and Zp3. However, *Zp4* is transcribed in *Mus musculus* oocytes but lacks a protein product due to the presence of several stop codons in its open reading frame (ORF) (Lefièvre et al., 2004; Evsikov et al., 2006; Goudet et al., 2008). Moreover, mass spectrometry analysis has failed to identify this protein (Boja et al., 2003).

Ultrastructural evidences suggest that mouse ZP is composed of filaments. Three different models were described; the first one suggests a filamentous structure where Zp2-Zp3 heterodimers are the basic repeating units of the filaments with cross-linking of filaments by dimeric Zp1 (Greve and Wassarman, 1985; Wassarman, 1988). The second one proposed by Dean in 2004, describes a ZP formed by repeats of Zp3-Zp2 and Zp3-Zp1 heterodimers that form the main fibrillar structure, being bound through the glycoproteins Zp1 and Zp2 (Dean, 2004). The third one, is a variation of the first model, so that the Zp1 glycoprotein is incorporated into the long filaments through its ZP domain; therefore in the mouse, the ZP would be formed by a fibrillar framework constituted by long polymers of Zp1-Zp2-Zp3 which are joined to each other by Zp1 homodimers through disulfide bonds forming a three-dimensional structure (Monné and Jovine, 2011; Stsiapanava et al., 2020). However, the ZP structure of the species with four proteins remains unproven.

The house mouse (*Mus musculus*) is an index species for biomedical research, and has been used as a model to study the ZP for more than 35 years (Liu et al., 1996; Rankin et al., 1996, 1999, 2001; Baibakov et al., 2012; Avella et al., 2014). Nevertheless, its ZP composition markedly differs from that seen in other mammals, including human. Thus, the lack of a good experimental animal model is one of the major hurdles to fully understanding the functional role of human ZP proteins (Gupta, 2018). Since rat (*Rattus* genus) has four glycoproteins and the house mouse (*Mus* genus) only three, *Zp4* was probably lost after their divergence around 12 MYA (Jaeger et al., 1986; Jacobs et al., 1989, 1990; Goudet et al., 2008). In order to determine more precisely when this pseudogenization took place, the *Zp4* gene was sequenced in different species of rodent that belong to the same group as *Mus* and *Rattus*, the Murinae subfamily, involving a particularly comprehensive taxonomic sampling within the genus *Mus* (Musser and Carleton, 2005). This study permitted better understanding of the unusual composition of the mouse ZP and has led to the proposal of a new animal model for studying human ZP. In order to ascertain whether the presence of four ZP glycoproteins in its composition could affect fertilization, an *in vitro* heterologous fertilization study using closely-related murine species was performed.

## Results

### Pseudogenization of *Zp4* Occurs Only in *Mus*

Our aim was to amplify and sequence the region of genomic DNA encompassing the exons 1–9 of the *Zp4* gene in several species of the subfamily Murinae (Table 1). Sequences were aligned to the corresponding genomic portions of DNA of *Mus* (chromosome 13) and *Rattus* (chromosome 17). Sequences of the mRNA obtained from *Mus mattheyi, Mus pahari*, and *Mastomys coucha* ovaries were added to determine the limits of the exons. The complete sequences (covering exons 1–9) were obtained only for *Rattus rattus* and *Mus minutoides*. For the other species, the coverage ranged between 70% (*Apodemus flavicollis* and *Mus minutoides*) and 13% (*Lemniscomys striatus* and *Malacomys longipes*).

**Table 1:** Muroid species and accession numbers of the sequences used for the phylogenetic study of the *Zp4* gene.

**Subfamily**	**Species**	**ZP4 (GenBank & Ensembl)**	**RNA (this study)**	**DNA (this study)**
Murinae	*Apodemus flavicollis*			LR990796, LR990797
Murinae	*Arviicanthis niloticus*			LR990798, LR990799
Murinae	*Grammomys surdaster*	XM_028769799		
Murinae	*Lemniscomys striatus*			LR990800
Murinae	*Malacomys longipes*			LR990801
Murinae	*Mastomys coucha*	XM_031359266	MH822871	
Murinae	*Maxomys whitheadi*			LR990802, LR990803, LR990804
Murinae	*Micromys minutus*			LR990805
Murinae	*Millardia meltada*			LR990806, LR990807
Murinae	*Mus caroli*	CAROLI_EIJ_v1.1 chr13/XM_021180912		LR990808, LR990809
Murinae	*Mus cookii*			LR990810
Murinae	*Mus crociduroides*			LR990811
Murinae	*Mus cypriacus*			LR990812, LR990813
Murinae	*Mus famulus*			LR990814
Murinae	*Mus macedonicus*			LR990815, LR990816
Murinae	*Mus mattheyi*		MH822867	
Murinae	*Mus minutoides*			LR990817, LR990818, LR990819
Murinae	*Mus musculus*	GRCm38 chr13/ENSMUST00000220980/NR_027813		
Murinae	*Mus pahari*	XM_029547650	MH822868	
Murinae	*Mus saxicola*			LR990820
Murinae	*Mus spicilegus*	MUSP714/ENSMSIT00000023447		LR990821, LR990822
Murinae	*Mus spretus*	SPRET_EiJ_v1 chr13		LR990823
Murinae	*Niviventer confucianus*			LR990824, LR990825
Murinae	*Otomys angoniensis*			LR990826, LR990827
Murinae	*Praomys jacksoni*			LR990828
Murinae	*Praomys tullbergi*			LR990829
Murinae	*Rattus exulans*			LR990830, LR990831
Murinae	*Rattus norvegicus*	AF456325		
Murinae	*Rattus rattus*	XM_032885241		LR990832
Gerbillinae	*Meriones unguiculatus*	XM_021663194/XM_021663196		
Cricetinae	*Cricetulus griseus*	XM_003505264/XM_027405493		
Cricetinae	*Mesocricetus auratus*	NM_001281648/DQ838550		
Arvicolinae	*Microtus ochrogaster*	XM_013353098		
Neotominae	*Peromyscus leucopus*	XM_028860421		
Neotominae	*Peromyscus maniculatus*	XM_006980166		
Spalacinae	*Nannospalax galili*	XM_00884135		

These new sequences were aligned with Zp4 sequences of other muroid rodents found in Genbank or ENSEMBL (Table 1). The full length alignment (exons 1–9) comprises 36 sequences and 5,140 bp (genomic DNA) and 1,301 bp (coding portion). Two portions of the sequences were scrutinized: the beginning of the gene (from exons 1–3) and the end of our alignment (exons 8 and 9), in both of which stop codons were found in *Mus musculus* (Goudet et al., 2008). The first fragment in all our samples was successfully amplified (except *Lemniscomys striatus*) and the second one in thirteen samples.

**Figure 1:**
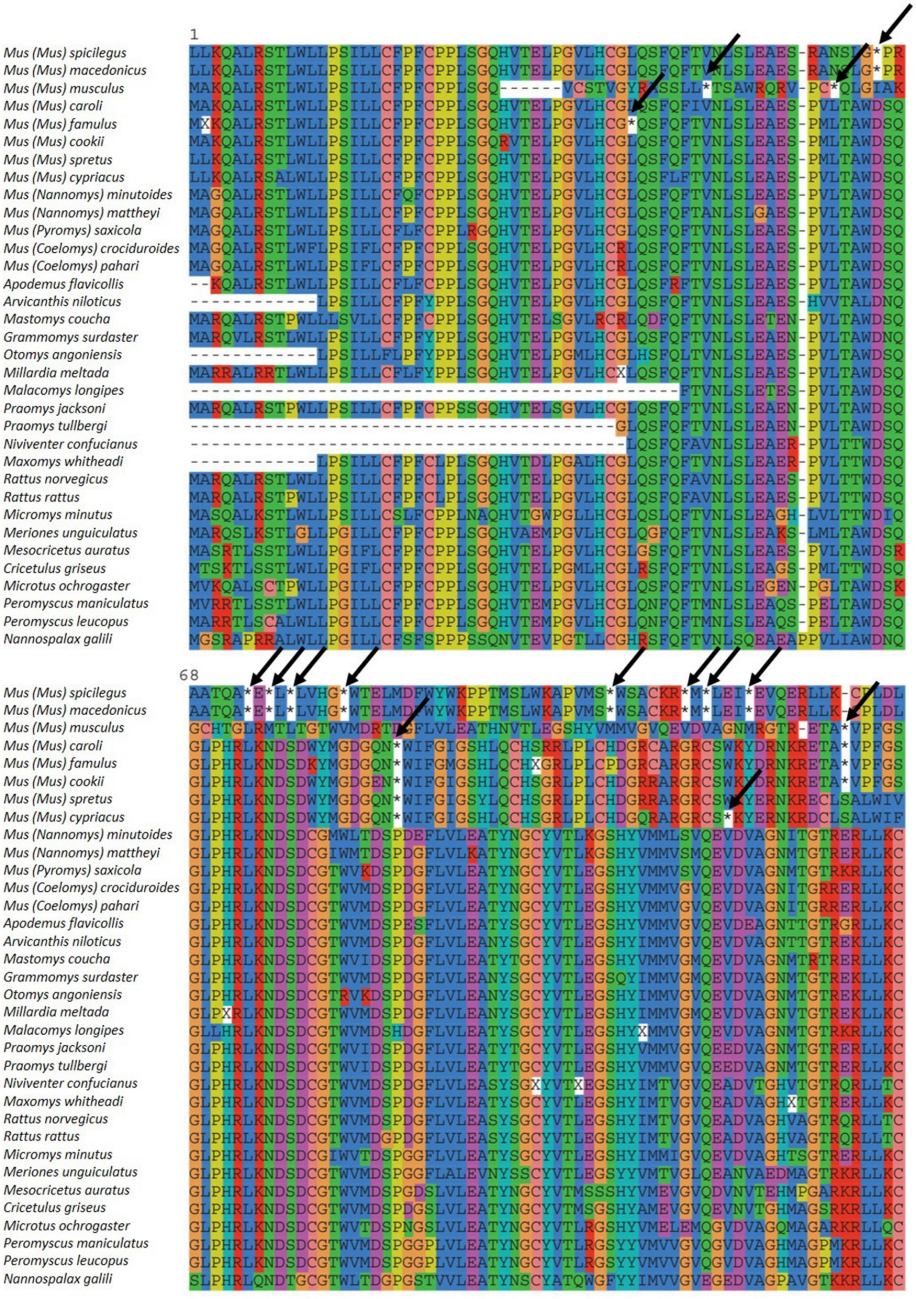
Zp4 alignment of the different species analyzed. Initial methionine is signaled with 1. Stop codons are marked by an asterisk (*) and arrows.

The results showed that stop codons are present in the first three exons of *Zp4* in eight species of the subgenus *Mus: M. caroli, M. cypriacus, M. cookii, M. famulus, M. macedonicus, M. musculus, M. spicilegus*, and *M. spretus* (Figure 1); however, they were also present in exons 8 and 9 in *M. musculus* and *M. spretus*.

**Figure 2:**
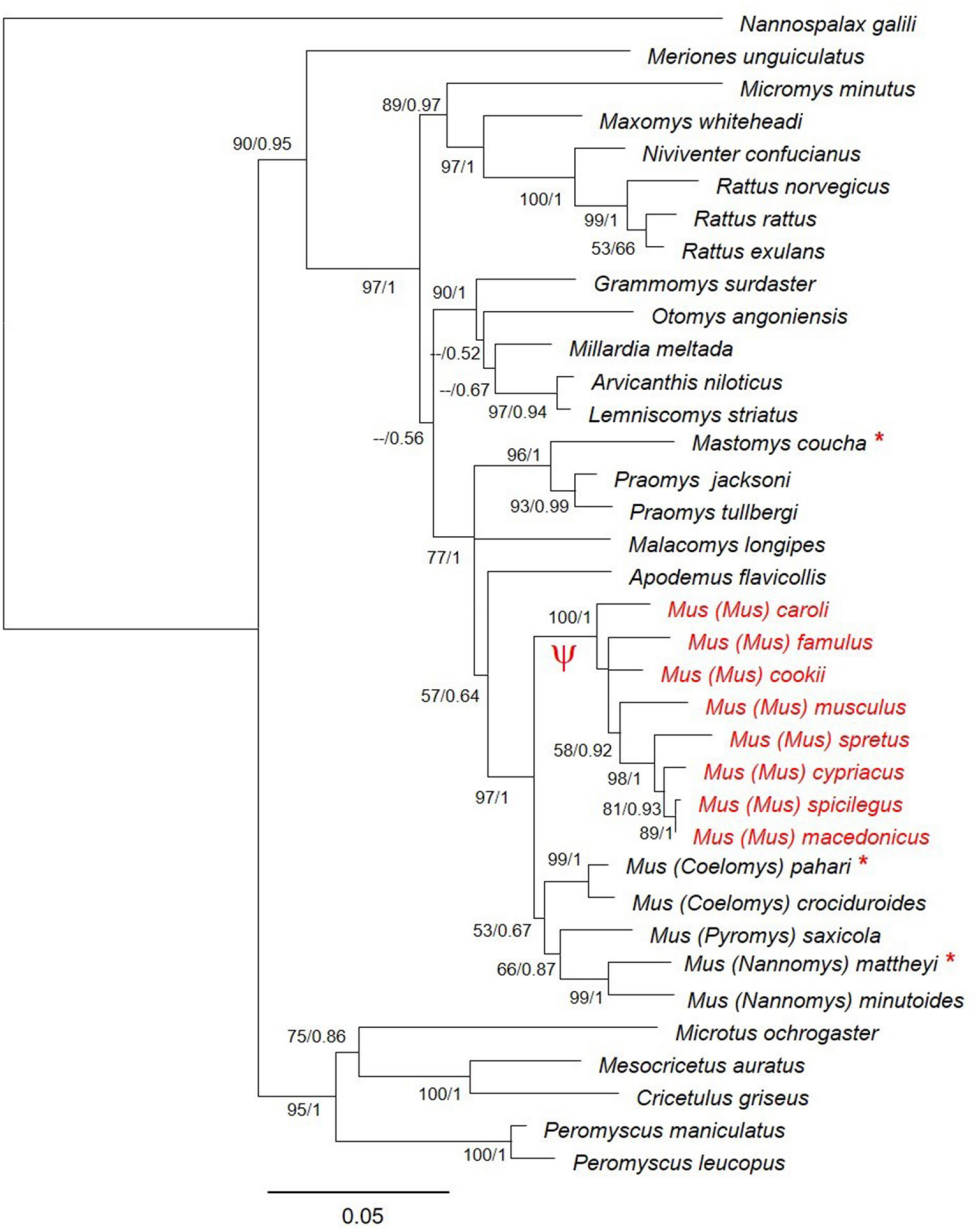
Phylogenetic relationships of *Zp4* gene in different species of muroid rodents. Bootstrap support and posterior probabilities are indicated for each node. The species where *Zp4* is a pseudogene are indicated in red. The symbol Ψ indicates the branch of the tree where the pseudogenization probably took place. The species for which we sequenced the mRNA are indicated with an asterisk.

The phylogenetic tree (Figure 2) confirms the monophyly of this subgenus and indicates that the pseudogenization took place after the divergence of the subgenus *Mus* and before species diversification. Previous studies reported that the four subgenera of *Mus* diverged between 6 and 7 MYA (Lecompte et al., 2008; Pagès et al., 2012; Meheretu et al., 2015). Within the subgenus *Mus* the earliest offshoot is estimated to have appeared at around 5 MYA (Pagès et al., 2012), indicating that the pseudogenization took place between 5 and 7 MYA.

### Zp1, Zp2, Zp3, and Zp4 Are Expressed in *Mus* (*Coelomys*) *pahari, Mus* (*Nannomys*) *mattheyi*, and *Mastomys coucha* Ovaries

To determine whether *Zp4* pseudogenization affects only the subgenus *Mus*, it was necessary to confirm the expression of the four ZP genes and proteins in other subgenera belonging to the genus *Mus*, in our case *Nannomys* and *Coelomys* (Musser and Carleton, 2005; Pagès et al., 2012). Individuals of two species—*Mus mattheyi* (subgenus *Nannomys*) and *Mus pahari* (subgenus *Coelomys*)—were studied. Furthermore, a species from another genus of the *Murinae* subfamily, *Mastomys coucha*, was also analyzed. The species were selected according to their availability and phylogenetic interest for this study.

Using RT-PCR analysis, full-length cDNAs of *Mus mattheyi* and *Mus pahari Zp1* (Supplementary Figure 1) and *Zp4* (Supplementary Figure 2) were obtained from total RNA prepared from ovaries. The sequence analysis indicated that they have a complete coding region. The open reading frames (ORFs) encode polypeptides with a theoretical molecular weight of 68.61 and 59.51 kDa (*Mus mattheyi* Zp1 and Zp4) and 68.37 and 59.54 kDa (*Mus pahari* Zp1 and Zp4).

These genetic sequences would translate a predictive protein in both species with a high degree of similarity to ZP1 and ZP4 proteins of other mammals (Supplementary Figures 3, 4). In the N-terminal region, a signal peptide is present, whose peptidase cleavage site was predicted by means of the Bendtsen et al. (2004) algorithm. The C-terminal region corresponds to the transmembrane domain (TMD), and is followed by a cytoplasmic tail (Krogh et al., 2001). Moreover, a basic amino acid domain upstream of the TMD may serve as a consensus furin cleavage site (CFCS) (Arg-Arg-Arg-Arg/RRRR). The molecules have a conserved ZP domain, which is present in most sequences of envelope glycoproteins in many species. Upstream of the ZP domain, there is a trefoil domain, characteristic of ZP1 and ZP4 proteins, with six Cys residues, as reported for ZP proteins (Bork, 1993), and between the signal peptide and the trefoil domain a single ZP-N domain at their N-termini, as reported previously (Callebaut et al., 2007; Nishimura et al., 2019) (Supplementary Figures 3, 4). The presence of all these domains indicates that the Zp1 and Zp4 of these rodents could share an apparent similar molecular structure with other ZP proteins.

**Figure 3:**
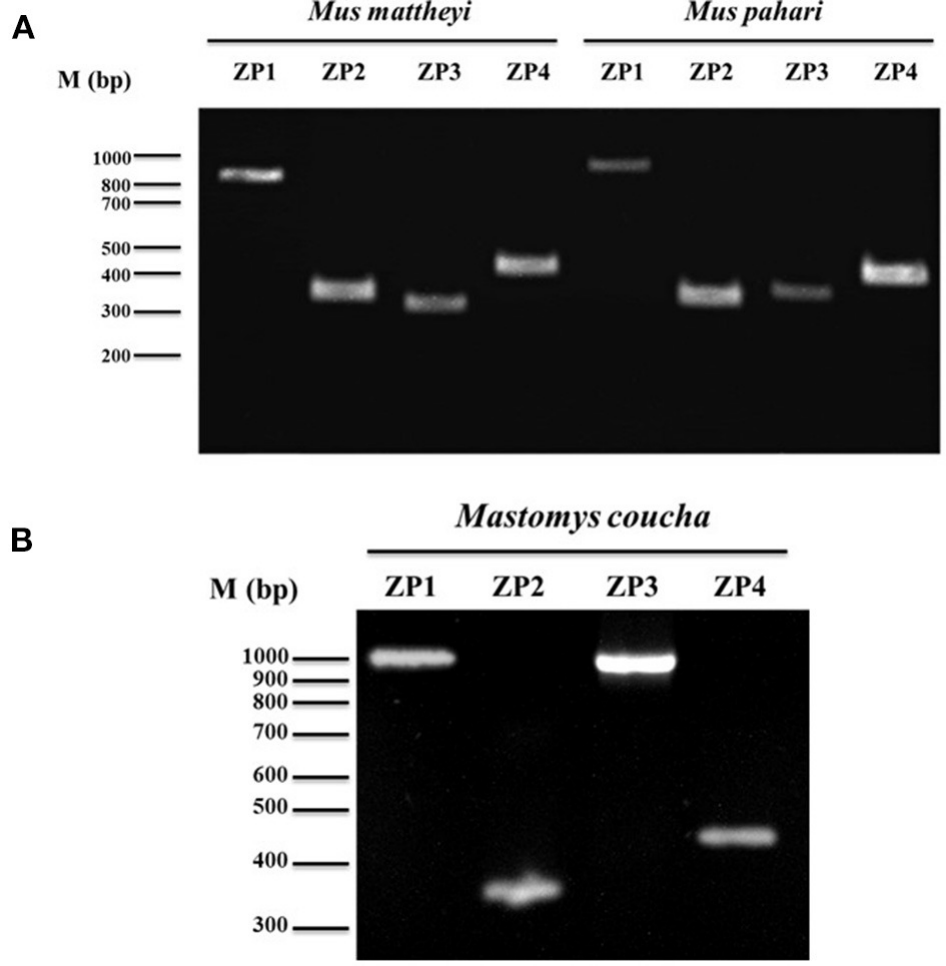
Analysis of *Zp1, Zp2, Zp3, and Zp4* gene expression in **(A)** *Mus mattheyi, Mus pahari* and **(B)** *Mastomys coucha* by RT-PCR. Amplicons corresponding to each gene are shown. Primers used for the amplifications of the different ZP genes are shown in Supplementary Table 2.

Thus, taking into consideration that ZP2 and ZP3 are present in all vertebrates described to date and that they have never suffered pseudogenization, the presence of a complete ORF of *Zp1* and *Zp4* mRNA in these murine species suggests a ZP consisting of four glycoproteins. Nevertheless, partial amplification of *Zp2* and *Zp3* were made in both species to demonstrate the presence of the four transcripts (Figure 3). Furthermore, *Zp1, Zp2, Zp3*, and *Zp4* mRNAs from *Mastomys coucha* were also partially amplified (Figure 3).

The mRNA of *Zp1, Zp2, Zp3*, and *Zp4* are effectively translated in proteins, as confirmed by the detection of several peptides belonging to Zp1 and Zp4 in *Mus mattheyi* and *Mus pahari* and the four proteins (Zp1, Zp2, Zp3, and Zp4) in *Mastomys coucha* (Table 2) by MS/MS analyses.

**Table 2:** Peptides identified by proteomic analysis in *Mus mattheyi, Mus pahari*, and *Mastomys coucha* ZPs.

**Peptides**	**Score**	**SPI**	**Sequence**	***n***
***Mus mattheyi***				
**ZP1**				
HIPCMVKGSPKEACQQAGCCYDSAK	3.26	58.6	234–258	2
GDNYRTQVVAtDK	3.37	90.6	474–486	3
**ZP4**				
*GSHYVMMVSMQEVDVAGNMTGTRER*	5.68	78.1	105–129	12
FSIAVSRNATSPPLRLDSLHLVFR	4.31	50.6	202–225	1
RRKSELHFETTTSISSkGPLILLQATK	3.48	56.2	471–497	1
LLKCPLDLRAPDAPSAEVCSPVPVK	3.54	64.2	130–154	1
DKSYGSYYGSDAYPLVK	3.38	63.6	331–347	1
KSELHFETTTSISSKGPLILLQATK	7.17	57.5	473–497	2
TSSPFPSHHQRFSIDTFSFMSAVR	7.03	56.8	412–435	1
*FSIDTFSFMSAVREK*	5.04	64.9	423–437	1
TQPGPLSLELQIAKDK	3.74	58.6	317–332	1
RKSELHFETTTSISSKGPLILLQATK	3.60	54.1	472–497	1
CTREGRFSIAVSRNATSPPLR	4.30	50.9	196–216	1
CTREGRFSIAVSR	4.28	86.1	196–208	1
***Mus pahari***				
**ZP1**				
FTIATFTLLDSSSQNALR	4.58	62.5	498–515	1
*SGYFTLVVSQETALTHGVMLDNVR*	5.73	70.7	275–298	2
**ZP4**				
*CPVDLHTTDASNAEVCSPVPVK*	6.75	60.2	133–154	3
LLKCPVDLHTTDASNAEVCSPVPVK	3.34	55.1	130–154	1
*AVYENELVAIRDVQAWGRSSITR*	5.91	70.9	260–282	1
ERLLKCPVDLHTTDASNAEVCSPVPVKER	3.44	60.8	128–156	2
*RIPVQKASSPFPSHHQRFSIA*	5.39	62.8	406–426	4
RERLLKCPVDLHTTDASNAEVCSPVPVK	4.03	52.8	127–154	1
***Mastomys coucha***				
**ZP1**				
*QAVLPNGRVDTAQDVTLICPKPDRIVTRDPYLAPPTTPEPFTPHTFALHPIT*	13.63	81.6	109–160	2
*GLAGPTVPHPQWGTLEPWELTEMDSV*	11.05	71.2	195–220	1
*EPWELTEMDSVGTHLPQERCRVASGHIPCMVKGS*	11.01	66	210–243	1
*VPHPQWGTLEPWELTEMDSVGTHLPQERC*	10.76	66.3	201–229	1
*MALTHGVMLDNVHLAYAPNGCPPTQ*	10.42	67.7	287–311	1
IATFTLLDSSSQNALRGQVYFFCSASACHPVGSKTCSTTCD	9.25	58.1	501–541	1
*CDSGIARRRRSSGHHNS*	7.42	88	540–556	1
*FTIATFTLLDSSSQNALRGQVYFFCSASACHPVGSKTCSTTCDSGIARRRR*	8.73	67.4	499–549	1
*RSSGHHNSTIQALNIVSSPGAVGFEDAAKLEPSGSSRNSSSR*	7.7	63.1	549–590	2
*GQVYFFCSASACHPVGSKTCSTTCDSGIARR*	7.4	66.6	517–547	1
*RRSSGHHNSTIQALNIVSSPGAVGFEDAAKLEPSGSSR*	6.16	66.8	548–585	1
SSGHHNSTIQALNIVSSPGAVGFEDAAKLEPSGSSR	5.57	57.5	550–585	1
**ZP2**				
*LNAYIKSHSSPVASVKPGPLQLVLQTYPDKS*	12.38	78.2	467–497	1
*TVVTSMNSLSLPQSA*	12.28	88.9	27–41	1
*PCGRSIYRLLSLLFTVVTSMNSLSLPQSANSAFPGTLICDKDGVRVEF*	12.2	71.1	13–60	1
*MDPNSYGITKDIIAKDIASKTLGAVAALVGLAVVIGF*	11.86	75.2	664–700	1
*TFSSKAICVPDLSVACNATHMTLTIPEFPGKLKS*	11.71	79.1	248–281	1
*KFPYKTCTLKVIGGYQMNIRVGDTS*	11.28	83.2	96–120	1
*TCTLKVIGGYQMNIRVGDTSTDVRGKDDMHHFFCPAIQAEAHETSEIVVCMEDLISFSFPQ*	11.28	88.2	101–161	1
QFYLSSLKLTFYFQGDMVSTVIDPECHCESPVSIDELCAQ	11.12	43.3	328–367	1
*SLDSPLCSVTCPAPLRSKREASKDDTMTV*	11.01	65.1	617–645	1
*QGDMVSTVIDPECHCESPVSIDELCAQDGFMDFEVYSHQTKP*	10.73	69	341–382	1
*WENPPSNIVFRNSEFR*	10.69	62.9	238–453	1
*KITFSSKAICVPDLSVACNATHMTLTIPE*	10.68	75.8	246–274	1
*HSSPVASVKPGPL*	10.36	88.2	474–486	1
*SMNSLSLPQSANSAFPGTLICDKDGVRVEFSSRFDMEKWNPAVVDTFGNEILNCTYAL*	10.33	82.9	31–88	1
*DDTMTVSLPGPILLLSDDSSSKGV*	10.12	69.2	640–663	5
*SSYLYTVQLKLLFSIPGQKITF*	10.02	69.8	228–249	1
*DDTMTVSLPGPILLLSDDSSSKGVMDPNSYGI*	9.36	62.3	640–671	1
*NSLSLPQ*	7.51	67.7	33–39	1
*SLDSPLCSVTCPAPLRSKREASKDDTMTVSLPG*	7.5	100	617–649	1
*IDSQKITLHVPANATGVAHYVQESSYLYTVQLK*	5.73	92.6	205–237	1
*LLSLLFTVVTSMNSLSLPQSANSAFPGTLICDKDGVRVEFSSR*	10.98	65.9	21–63	1
*NDPNIKLALDDCWATSSEDPASVPQWQIVMDGCAYELDNYR*	9.98	87.6	527–567	2
*DEYPVVRYLRQPIYMEVTVLNRNDPNIKLALDDCWATSSEDPASVPQWQIVMD* *GCAYELDNYR*	9.68	68.4	505–567	1
*VQSLGLARFHIPLNGCGTQQKFEGDKVIYENEIHGLWENPPSNIVFRNSEFR*	9.14	83.1	402–453	1
VIYENEIHGLWENPPSNIVFRNSEFRMTVR	8.8	44.1	428–457	3
*REASKDDTMTVSLPGPILLLSDDSSSKGVMDPNSYGITKDIIAK*	8.55	61.3	635–678	1
VEFSSRFDMEKWNPAVVDTFGNEILNCTYALDMEK	8.26	34.1	58–92	1
*REASKDDTMTVSLPGPILLLSDDSSSK*	7.91	62.2	635–661	1
*MARWQRKESVNPPCGRSIYRLLSLLFTVVTSMNSLSLPQSANSAFPGTLICDK* *DGVRVEFSSRFDMEK*	7.86	70.5	1–68	1
*ITFSSKAICVPDLSVACNATHMTLTIPEFPGK*	7.84	83.9	247–278	1
*DEYPVVRYLRQPIYMEVTVLNR*	7.54	54.4	505–526	1
*FILKFPYKTCTLKVIGGYQMNIRVGDTSTDVR*	7.47	69.8	93–124	1
TFAFVSEARRLNSLIYFHCSALICNQVSLDSPLCSVTCPAPLR	6.95	48.1	590–632	1
*SKREASKDDTMTVSLPGPILLLSDDSSSKGVMDPNSYGITK*	6.79	62	633–673	1
*DDTMTVSLPGPILLLSDDSSSKGVMDPNSYGITKDIIAKDIASK*	5.9	85.2	640–683	1
*SKREASKDDTMTVSLPGPILLLSDDSSSKGVMDPNSYGITKDIIAK*	5.83	85.6	633–678	1
*LADENQNVSEMGWIIKIGNGTR*	5.5	80.8	166–187	6
TTFHSAGSSVAHSGHYQRFDVK	4.3	87.1	568–589	1
**ZP3**				
*FRATVSSEEKLAFSLRLMEENWNTEKSSPTFHLGEVAHLQAEVQTGSHL* *PLQLFVDYCVATPSPAPD*	12.86	66.9	149–215	1
*VDSHGCLVDGLSESFSAFQVPRPRPEMLQFTVDVFHFANSSRNTLYITCHL*	8.36	72	225–275	1
*TVGPLIVLGNANDQTVEGWTSSAQTSMALGLGLVTMAFLTLAA*	6.39	94.6	352–394	1
*ITCHLKVAPANQIPDKLNKACSFNKTSQSWLPVEGDADICDCCSH*	5.5	100	271–315	1
**ZP4**				
*QEVDVAGNMTRTRERLLKCPL*	12.46	64.1	115–135	1
*GGQVYLHCSASVCQPAGMPSCMIICPASRRRRKSELYFENT*	12.25	33.7	443–483	1
*PSPISRGDCEEVGCCYSSEEEEAGSCYYGNTVTSHCTREGGFSI*	11.98	66.4	154–206	1
*GSHYVMMVGMQEVDVAGNMTRTRERLLKCPLDLPSKAPDAPSAEVCSPVPIKERL*	11.63	70.6	105–159	1
*QPAGMPSCMIICPASRRRRKSELYFENTTS*	11.13	74.6	456–485	1
*TREGGFSIVVSRNATSPPLRLDSLRLVSR*	10.6	59.7	199–227	1
*VNIRVLALPPPIPKTQPGPLS*	8.68	61.5	305–325	1
*QVLGGQVYLHCSASVCQPAGMPSCMIICPASRR*	8.69	60	440–472	2
*LVSRNNSGCDPVMTTSTFVLFQFPFSSCGTTRRITGDQALYENELVAIQDVQA* *WGRSSITRDSNFRLR*	8.63	80	224–291	1
QVLGGQVYLHCSASVCQPAGMPSCMIICPASR	7.2	36.7	440–471	1
*GDCEEVGCCYSSEEEEAGSCYYGNTVTSHCTREGGFSIVVSR*	6.87	81.6	169–210	1
*LDSLRLVSRNNSGCDPVMTTSTFVLFQFPFSSCGTTRR*	6.84	51.1	219–256	1
*QRFSIATFSFMSAVR*	5.41	75.4	423–437	2

*Peptides with a score higher than 5 and percentage-scored peak (SPI) intensity of 60%, which are the threshold criteria for a positive identification, are shown in italics. “n” represents the number of times that the peptide has been detected*.

**Figure 4:**
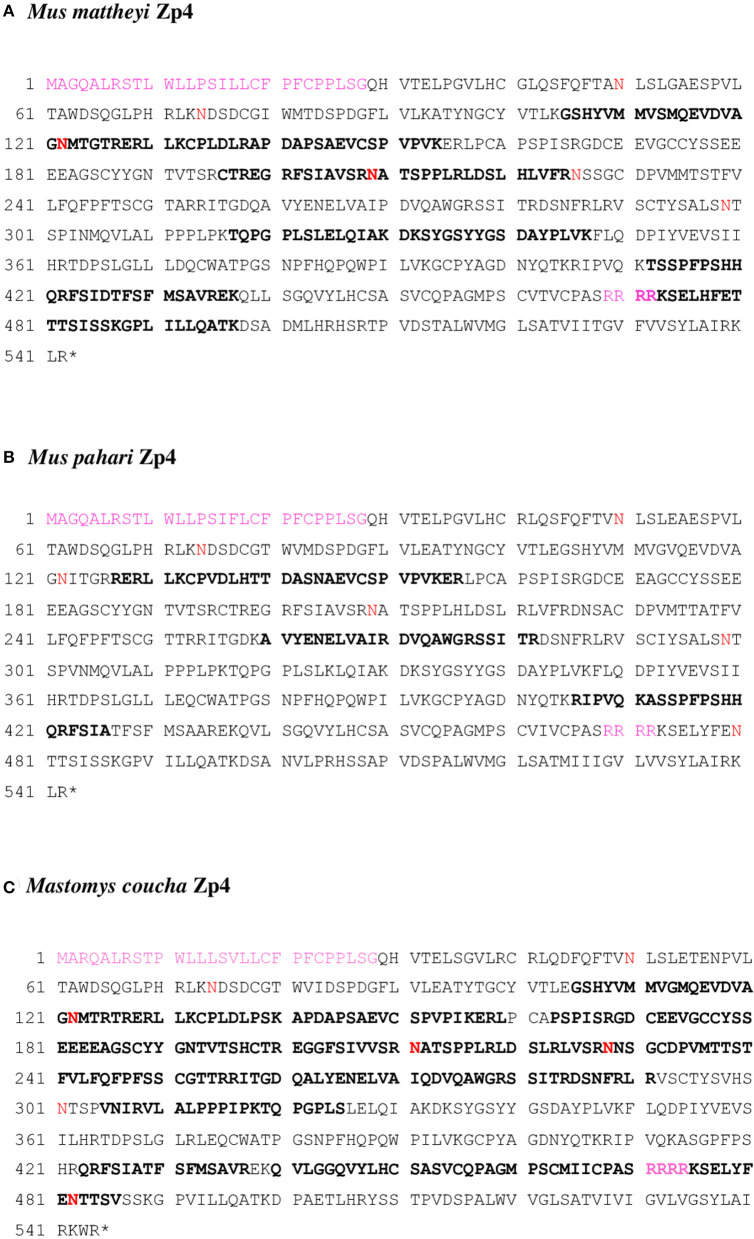
**(A)** *Mus mattheyi* Zp4 (AYN07267.1), **(B)** *Mus pahari* Zp4 (AYN07268.1), and **(C)** *Mastomys coucha* Zp4 (XP_031215126.1) amino acid sequences. Bold sequences are the tryptic peptides obtained by MS/MS. The putative N-glycosylation sites are in red. The signal peptide and the furin cleavage site (Arg-Arg-Arg-Arg) are shown in pink.

A total of 12, 6, and 13 different peptides corresponding to Zp4 were identified in the different analyses, yielding a sequence coverage of 30.99, 16.74, and 49.08% for *Mus mattheyi, Mus pahari*, and *Mastomys coucha*, respectively (Figure 4).

Taken together, these data indicate that four ZP proteins are expressed in *Mus mattheyi, Mus pahari*, and *Mastomys coucha* ovaries.

### Heterologous *in vitro* Fertilization (Oocyte With Three ZP Proteins *vs*. Four ZP Proteins)

The next question was whether the ZP composition of the egg could interfere with fertilization, for this reason we performed *in vitro* fertilization experiments with species differing in their ZP composition. Four rodents with different ZP composition were used: *Mus musculus* with three ZP proteins (Zp1, Zp2, and Zp3) and *Mus mattheyi, Mus pahari*, and *Mastomys coucha* with four ZP proteins (Zp1, Zp2, Zp3, and Zp4). The *in vitro* fertilization rates in a non-competitive context were analyzed (Table 3).

**Table 3:** Results of cross *in vitro* fertilization (with cumulus and without cumulus cells).

	**Percentage of fertilization with spermatozoa of**			
**Oocytes of**	***Mus musculus* (*n =* 6)**	***Mus pahari* (*n =* 3)**	***Mus mattheyi* (*n =* 2)**	***Mastomys coucha* (*n =* 2)**
*Mus musculus* (*n =* 26)	79.16 (*n =* 216)	6.1 (*n =* 98)	3.22 (*n =* 93)	67.14 (*n =* 70)
*Mus pahari* (*n =* 15)	67.5 (*n =* 40)	3.5 (*n =* 28)	0 (*n =* 7)	–
*Mus mattheyi* (*n =* 12)	11.7 (*n =* 34)	0 (*n =* 2)	0 (*n =* 6)	–
*Mastomys coucha* (*n =* 4)	0 (*n =* 44)	–	–	0.81 (*n =* 123)
	**Percentage of fertilization with spermatozoa of**			
**Oocytes with cumulus cells of**	***Mus musculus***	***Mus pahari***	***Mus mattheyi***	***Mastomys coucha***
*Mus musculus*	81.25 (*n =* 160)	8.70 (*n =* 46)	4.76 (*n =* 63)	67.14 (*n =* 70)
*Mus pahari*	56.52 (*n =* 23)	0 (*n =* 15)	–	–
*Mus mattheyi*	11.73 (*n =* 34)	0 (*n =* 2)	0 (*n =* 6)	–
*Mastomys ocucha*	0 (*n =* 44)	–	–	0.81 (*n =* 123)
**Oocytes without cumulus cells of**				
*Mus musculus*	73.21 (*n =* 56)	3.85 (*n =* 52)	0 (*n =* 30)	–
*Mus pahari*	82.35 (*n =* 17)	7.69 (*n =* 13)	0 (*n =* 7)	–
*Mus mattheyi*	–	–	–	–

*First part of the table is a summary of the oocytes used with and without cumulus cells*.

Oocytes from the four species were co-incubated with spermatozoa in conspecific or heterospecific reciprocal crosses. Fertilization success in the conspecific crosses was high only in *Mus musculus* (79.16%), whereas in the other species the rate was zero or very low (0% in *Mus mattheyi*, 0.81% in *Mastomys coucha*, and 3.5% in *Mus pahari*). When *Mus musculus* spermatozoa participated in the fertilization the rates were 67.5% in co-incubation with ova from *Mus pahari*, 11.7% with ova from *Mus mattheyi*, and 0% with ova from *Mastomys coucha*. The fertilization rate was very low when *Mus mattheyi* or *Mus pahari* spermatozoa were used, except for *Mastomys coucha* spermatozoa with *Mus musculus* oocytes (67.14%). Our observations showed that *Mus mattheyi* and *Mus pahari* sperm were still able to adhere to the heterologous ZP, but we observed that the number of spermatozoa that adhere to the ZP was much lower than in the control group. Besides, we observed that when the sperm adhered to the ZP, they remained immobile (data not shown). Taking into consideration the results obtained in the *in vitro* fertilization crosses between *Mus musculus vs*. *Mus pahari* (67.5%) and *Mastomys coucha vs*. *Mus musculus* (67.14%), it can be concluded that the presence of the 3 or 4 ZP proteins is not a limiting factor for *in vitro* fertilization.

### Analysis of ZP4 Positive Selection

Genes with a role in fertilization show a common pattern of rapid evolution, which can be attributed to positive selection. An analysis of such selection in *Zp4* gene was made in order to know the level of interspecific divergence and the existence of positive selection sites.

The selection analysis pointed to significant positive selection for both muroid datasets. For the dataset of the 28 muroids, both tests (M1a *vs*. M2a and M7 *vs*. M8) were significant (*p* < 0.05 in the former case and *p* < 0.01 in the latter), and one site (141 R) showed a posterior probability > 95% (Table 4). For the dataset of the 13 muroids, both tests (M1a vs. M2a, M7 vs. M8) were significant (*p* < 0.01), and one site (547 L) showed a posterior probability > 95% (Table 4).

**Table 4:** Results of maximum likelihood models of Zp4 of muroid rodents.

**Model code**	**Log-likelihood**	**Parameters estimates**	**Positively selected sites (BEB)**
28 sequences, 711 sites			
M1a (NearlyNeutral)	−4431.388215	*p_0_ =* 0.66320, *w_0_ =* 0.18102, *w_1_ =* 1	
M2a (PositiveSelection)	−4427.068269	*p_0_ =* 0.65668, *p_1_ =* 0.33691, *w_0_ =* 0.18357, *w_1_ =* 1, *w_2_ =* 4.86401	**141 R**, 225 S
M7 (beta)	−4435.034712	*p =* 0.56075, *q =* 0.72347	
M8 (beta&ω>1)	−4429.555890	*p_0_ =* 0.99203, *p =* 0.60009, *q =* 0.78536, *w_*s*_ =* 4.35054	21 P, 57 S, 124 M, **141 R**, 225 S
13 sequences, 1,644 sites M1a (NearlyNeutral)	−8045.827139	*p_0_ =* 0.55934, w_0_ = 0.12004, w_1_ = 1	
M2a (PositiveSelection)	−8040.634099	*p_0_ =* 0.55158, p_1_ = 0.43982, w_0_ = 0.12076, w_1_ = 1, w_2_ = 5.14829	141 R, 293 R, 419 S, 439 V, 444 L, 547 L
M7 (beta)	−8048.563689	*p =* 0.29653, *q =* 0.31182	
M8 (beta&ω1)	−8042.092294	*p_0_ =* 0.99002, *p =* 0.31394, *q =* 0.33456, *w_*s*_ =* 4.73038	4 Q, 43 Q, 57 S, 105 K, 112 M, 115 M, 123 N, 127 T, 141 R, 145 A, 146 P, 147 S, 156 V, 169 R, 198 R, 204 R, 219 R, 239 M, 274 P, 277 Q, 281 R, 293 R, 339 G, 418 S, 419 S, 439 V, 444 L, 467 V, 468 T, 506 A, 508 M, 514 R, 515 T, 517 V, 525 M**, 547 L**

*The numbers in bold correspond to positively selected sites with P > 95%*.

## Discussion

Our results indicate that the pseudogenization of *Zp4* in mice is a relatively recent event that took place during the evolution of the genus *Mus*, a genus that encompasses more than 40 species divided into four subgenera: *Mus, Pyromys, Nannomys*, and *Coelomys* (Musser and Carleton, 2005; Shimada et al., 2010; Suzuki and Aplin, 2012). The subgenus *Mus* is by far the most extensively studied as it includes the cosmopolitan commensal *Mus musculus* (the house mouse), which has been used as a model to study the ZP and gamete interaction for the last four decades. This subgenus comprises 14 species (Auffray and Britton-Davidian, 2012). The other subgenera are *Nannomys*, the African pygmy mouse with 19 recognized species, and two South-East-Asian subgenera *Coelomys*, the shrew mouse with four species, and *Pyromys* the spiny mouse with five species (Musser and Carleton, 2005).

DNA sampling among the genus *Mus* included species belonging to the four subgenera, and the mRNA and protein analyses included species of two of these four subgenera for which no data had previously been available (*Nannomys* and *Coelomys*).

### Analysis of Zp4 Protein in *Mus (Nannomys) mattheyi* and *Mus (Coelomys) pahari*

The DNA and mRNA analyses of *Mus mattheyi* and *Mus pahari Zp4* sequences indicated the presence of a coding sequence for a full-length protein. The alignment showed a high degree of conservation: 87.32 and 88.05% with *Mastomys coucha*, 76.47 and 77.35% with hamster, 82.94 and 83.49% with rat, and 63.82 and 64.38% with human, for *Mus mattheyi* and *Mus pahari*, respectively. At the ZP domain, the 10 cysteines found were conserved in all the species. The furin cleavage site, described in human (Kiefer and Saling, 2002) and rat (Hoodbhoy et al., 2005) coincided with the potential sites for the rodents analyzed (Supplementary Figure 4).

Six (Asn50, Asn74, Asn122, Asn209, Asn226, and Asn299) and five (Asn50, Asn74, Asn122, Asn209, and Asn299) potential N-glycosylation sites were identified in the mature protein in *Mus mattheyi* and *Mus pahari*, respectively. Of which, Asn122 and Asn209 were identified by proteomics in *Mus mattheyi*, indicating that these sites are not glycosylated or not always occupied (Figure 4). The potential N-glycosylation sites Asn50, Asn74, and Asn226 have been identified in rat Zp4 (Hoodbhoy et al., 2005), so they seem to be conserved in Murinae; however, Asn299 has not been detected in the rest of the species analyzed, implying that there are differences in the glycosylation pattern between these species and the rat. Further studies are necessary to identify the glycosylation sites and the type of oligosaccharide chain present.

In mature Zp4 protein, a total of 76 and 73 potential O-glycosylation sites were found in *Mus mattheyi* and *Mus pahari*, respectively. Some of the peptides identified contained some of these O-glycosylation sites, 25 in *Mus mattheyi* and 11 in *Mus pahari*, so that they would be free or partially occupied in the mentioned proteins (Figure 4). O-glycosylation data are only available for sow and rat ZP4 (Kudo et al., 1998; Hoodbhoy et al., 2005). An O-glycosylated region (Thr296, Ser298, Ser301, Ser304, and Thr312) has been described in rat (Hoodbhoy et al., 2005), and is conserved in *Mus mattheyi* and *Mus pahari* (Ser298, Ser301, and Ser304), in addition to Thr296, which is only present in *Mus mattheyi* (Supplementary Figure 4).

Taking into account that HPLC/MS analysis can be considered a semi-quantitative technique, the fact that the coverage for Zp1 in *Mus mattheyi* and *Mus pahari* was 7.22 and 7.98%, respectively, compared to the coverage for Zp4 of 30.99% in *Mus mattheyi* and 16.74% in *Mus pahari*, this suggests that Zp1 is less abundant in the ZP than Zp4. These results coincide with those published by Boja et al. (2003) for house mouse (ZP coverage of 56% for Zp1, 96% for Zp2 and 100% for Zp3), and for rat with a protein coverage of 52% for Zp1 and 70% for Zp4 (Boja et al., 2005; Hoodbhoy et al., 2005). They would also agree with the results published for other species, such as rabbit (Stetson et al., 2012) and cat (Stetson et al., 2015), where the coverage of ZP1 is lower than that of ZP4; however, they do not coincide with the results published for hamster, where the coverage of ZP1 was 12.6% and that of ZP4 11.2% (Izquierdo-Rico et al., 2009). Future studies using quantitative proteomics are needed to clarify the ZP protein ratios in different species.

### Evolution of ZP Proteins in Muroid Rodents

The DNA analysis in different taxa showed the presence of stop codons in the eight species belonging to subgenus *Mus* and no stop codons in the other *Mus* species. The gene expression analysis in *Mus pahari* (subgenus *Coelomys*) and *Mus mattheyi* (subgenus *Nannomys*) clearly demonstrated the presence of four transcripts (*Zp1, Zp2, Zp3*, and *Zp4*) using RT-PCR and four ZP proteins using proteomic analysis. These results agree with previous studies that reported the existence of four proteins in the ZP in other placental species like human (Lefièvre et al., 2004), rat (Hoodbhoy et al., 2005), hamster (Izquierdo-Rico et al., 2009), rabbit (Stetson et al., 2012), cat (Stetson et al., 2015), or ferret (Moros-Nicolás et al., 2018c). These four proteins were also present in some marsupials, even though in this group the evolution of the ZP proteins is more complex as there are several copies of the *ZP3* gene (Moros-Nicolás et al., 2018a). The four-protein composition could be considered as the ancestral state in eutherian mammals, and ZP1 or ZP4 being lost in some lineages during their evolution.

Until now the ZP composition in the Murinae subfamily was only known in the rat (with four glycoproteins) and house mouse (with three glycoproteins), suggesting that *Zp4* was lost after their divergence around 11.2 MYA (Aghová et al., 2018). Using comprehensive taxonomic sampling within the subfamily, especially within the genus *Mus* (with representative taxa of the four subgenera), we were able to narrowed down the approximate date of the loss of *Zp4*. First, all other murine genera included in our study seem to have a functional *Zp4*, suggesting a more recent loss meaning that it occurred within the genus *Mus*, which diverged around 7.2 MYA (Pagès et al., 2012). Second, we found that all *Mus* species from the *Coelomys, Nannomys* and *Pyromys* subgenera have four glycoproteins, while all species from the *Mus* subgenus have only three, suggesting that *Zp4* pseudogenized early in the *Mus* subgenus lineage. Previous studies have reported that the four subgenera of *Mus* diverged between 6 and 7 MYA (Chevret et al., 2005; Lecompte et al., 2008; Pagès et al., 2012), initially the subgenus *Coelomys*, then *Nannomys*, and finally *Mus* and *Pyromys* (Veyrunes et al., 2006). Within the subgenus *Mus*, the earliest offshoot is estimated to have appeared at around 5 MYA (Pagès et al., 2012), indicating that *Zp4* pseudogenization took place 5–7 MYA.

Pseudogenization events are not rare in the ZP family. *ZP1* has been identified as a pseudogene in several species (Goudet et al., 2008; Tian et al., 2009; Stetson et al., 2012; Moros-Nicolás et al., 2018c). The *ZPAX* gene was lost in mammals before the divergence between marsupials and placentals (Tian et al., 2009). These multiple and independent loss events may be explained in part as the consequence of a duplication event, since *ZP1* and *ZP4* are paralogue genes (Bausek et al., 2000). After duplication, three evolution events may occurred to the duplicate copies: (a) the ancestral function is partitioned and shared by both copies (subfunctionalization); (b) one gene acquires a new function and the other retains the original one (neofunctionalization), or (c) one gene conserves the original function and the other degenerates to a pseudogene (Cañestro et al., 2013).

The duplication took place in vertebrates before the mammalian divergence (Goudet et al., 2008), and after the duplication event, *ZP4* or *ZP1* may have pseudogenized in some mammalian lineages, while the remaining ZP protein continued to perform the function of the ancestral gene. In the house mouse, the pseudogenization of *Zp4* indicates that Zp1 retained the function of the ancestral gene.

The evolution of male and female reproductive proteins was probably promoted by positive Darwinian selection. Moreover, comparative sequencing studies among taxonomic groups have led to the discovery that reproductive proteins evolve more rapidly than other genes expressed in other tissues (Swanson et al., 2001; Torgerson et al., 2002). This positive selection has been described in seminal plasma proteins (Kingan et al., 2003; Dorus et al., 2004), oviductal proteins (Moros-Nicolás et al., 2018b), and also in other proteins related with fertilization, such as ZP3, CatSper1 or CD9 (Swanson et al., 2001, 2003) These proteins would have been under a selective pressure that may be related to male-female interaction, in this case, sperm-egg interaction. Our analysis identified two sites in Zp4 that are under positive selection (141R and 547L**)**. These results strongly suggest that *Zp4* gene has been subjected to positive selection during evolution. These amino acids appear to be important for the protein function. Future studies using direct mutagenesis will be useful to unravel the specific function of these amino acids in ZP4 protein.

### Functional Implication of the Presence of ZP4 in the ZP Matrix

Several studies have analyzed the function of different ZP proteins. Recent studies in human demonstrate that mutations in *ZP1* gene are related to infertility (Huang et al., 2014; Sun et al., 2019; Yuan et al., 2019), these mutations could affect the shuttling of glycoproteins to the secretory pathway, which would prevent the formation of the ZP around the ova, but also the formation and development of eggs (Huang et al., 2014; Sun et al., 2019; Yuan et al., 2019). The house mouse has provided interesting information on the functions of the different ZP proteins thanks to the use of animals genetically modified as KO and transgenic (Liu et al., 1996; Rankin et al., 1996, 1999, 2001; Dean, 2004). It was demonstrated that Zp1 offers stability and structural integrity to the matrix. KO mice for *Zp1* have an abnormal ZP, being more porous; however, these mice are fertile, although their litter sizes are low (Rankin et al., 1999). On the other hand, KO mice for *Zp2* or *Zp3* present oocytes that are not surrounded by a ZP (Liu et al., 1996; Rankin et al., 1996, 2001). In the case of rodents with four ZP proteins, the roles played by Zp1 and Zp4 remain unresolved.

The study of ZP4 was not possible until now in animal models because, while the KO technology was well-developed in the house mouse (*Mus musculus*), which has a pseudogenized *Zp4*, the technique was very difficult to perform in rat, hamster and rabbit. However, development of CRISPR-cas9 technology made it possible to develop the KO technique in species that possess ZP4 (Fan et al., 2014; Bae et al., 2020). In fact, we have recently reported the phenotype of the female rabbit without the *ZP4* gene (Lamas-Toranzo et al., 2019). The female rabbit is subfertile and it was observed that this protein is crucial for the embryo development but not for fertilization (Lamas-Toranzo et al., 2019). Moreover, the ZP was significantly thinner, more permeable, and exhibited a more disorganized and fenestrated structure suggesting a structural role (Lamas-Toranzo et al., 2019). The development of KO animals for *ZP4* in other species with four ZP proteins, like the rat or the mice presented in this work could also be a useful tool to study the function of this gene. Furthermore, transgenic mice showing a humanized ZP4 have provided valuable information (Yauger et al., 2011). Indeed, these transgenic mice are fertile; however, their ZP is not recognized by human sperm, which means that ZP4 is not sufficient to support human sperm binding to the ZP (Yauger et al., 2011).

A previous study has shown that heterologous fertilization between different species of rodents is possible, although the success is directly related to the phylogenetic proximity of the species (Roldan et al., 1985): the heterologous fertilization rate *in vivo* and *in vitro* is considerably lower than the homologous fertilization rate (Roldan et al., 1985; Roldan and Yanagimachi, 1989; Dean and Nachman, 2009; Martín-Coello et al., 2009). Furthermore, in those cases in which embryo culture was carried out, cleavage arrest or embryo degeneration was observed (Roldan et al., 1985). In our study, heterologous IVF was possible when the spermatozoa from *Mus musculus* had to fertilize the oocytes from *Mus mattheyi* and *Mus pahari*, demonstrating that the presence of Zp4 is not involved in the species-specific binding of sperm. This also means that a ZP formed of four glycoproteins is neither a physical nor biological barrier for the spermatozoa of species with a ZP formed of three glycoproteins, at least in *in vitro* conditions, indicating that Zp4 does not produce any steric hindrance that impedes the specific gamete interaction.

## Conclusion

The present study provides new insights into the molecular evolution of Zp4 in rodents showing that *Zp4* pseudogenization is restricted to the subgenus *Mus*, which diverged around 6 MYA. The use of murine species with four ZP proteins may therefore be suitable for studying the structure and functionality of ZP proteins from most species, including humans. These rodents must be considered a tool of great value due to the possibility of applying transgenesis and KO techniques to study the function of these proteins and because of their short reproduction cycle compared to other species.

## Methods

### Ethical Approvals

All animal procedures were performed following the Spanish Animal Protection Regulation, RD 1201/2005 which conforms to European Union Regulation 2003/65. All animal experiments were approved by the institutional review board of the University of Murcia according to the guide for Care and Use of Laboratory Animals as adopted by the Society for the Study of Reproduction.

### Animals

Adult females and males of four species of murine rodents were used: the house mouse (*Mus musculus*), Matthey's mouse [*Mus mattheyi* (subgenus *Nannomys*)], Gairdner's shrewmouse [*Mus pahari* (subgenus *Coelomys*)], and the southern multimammate mouse Gairdner's shrewmouse (*Mastomys coucha*). *Mus musculus* specimens were of the hybrid strain C57CBAF1, purchased from Harlan Ibérica, (Barcelona, Spain), while *Mus mattheyi* and *Mus pahari* were obtained from the “Institut des Sciences de l'Evolution de Montpellier” (Montpellier, France) and *Mastomys coucha* specimens were obtained from Hobbyzoo (Madrid, Spain), whose species were verified by PCR amplification of the cytochrome b. Animals were kept under standard laboratory mouse conditions in an environmentally controlled room with a 14 h light:10 h darkness photoperiod under constant temperature and relative humidity conditions. Animals were provided with food (Harlan Ibérica, Barcelona, Spain) and water, both available *ad libitum*. Animals to be used for the experiments were weaned when they were 4 weeks old. Males were kept in individual cages and were used when they were >12 weeks old; after weaning, females were housed together. *Mus musculus* females were used when they were 6–8 weeks old, and *Mastomys coucha, Mus mattheyi*, and *Mus pahari* females were used when they were 6–12 weeks old.

### Collection of Mouse Ovaries

Ovaries were obtained from three different species of mouse: *Mastomys coucha, Mus mattheyi*, and *Mus pahari*. The animals were sacrificed by CO_2_ overdose, and the ovaries were obtained and frozen in liquid nitrogen and kept at −80°C (for molecular and proteomic analysis) or washed in PBS and used directly (to obtain the ZPs).

### Collection of Mouse Oocytes

Before oocytes were obtained from *Mastomys coucha, Mus mattheyi, Mus musculus*, and *Mus pahari*, the animals were subjected to a hormonal treatment to induce superovulation. Females were injected intraperitoneally with 7.5 IU of equine Chorionic Gonadotrophin (eCG) (Sigma-Aldrich, St. Louis, USA), followed 48 h later by 5 IU of human Chorionic Gonadotrophin (hCG) (Lepori Pharma, Spain). The animals were sacrificed 14 h after hCG injection by cervical dislocation and their oviducts were removed. Cumulus-oocyte-complexes (COCs) were obtained from the ampulla of the uterine tube and placed in PBS (for proteomic analysis) or HTF medium (for IVF analysis), COCs were removed or not by gently pipetting into 0.5% hyaluronidase (Sigma-Aldrich, St. Louis, USA).

### Zona Pellucida Isolation in *Mastomys coucha*

To obtain the isolated ZPs, animals were subjected to ovarian stimulation and the oocytes were obtained as explained above. Cumulus cells (CCs) were removed by using 0.5% hyaluronidase (Sigma-Aldrich, St. Louis, USA) in PBS, and the ZPs were obtained after vigorous pipetting of each oocyte using a narrow-bore micropipette in PBS, followed by four washes in PBS to eliminate the oocyte debris. ZPs were solubilized for 30 min at 65°C (Accu Block^TM^, Labnet, USA), and kept at −20°C until use.

### *In vitro* Fertilization

As mentioned above females of the four species were subjected to a hormonal treatment to induce superovulation. *In vitro* fertilization was performed as previously described by Hourcade et al. (2010). Males were killed by cervical dislocation. The epididymides and vasa deferentia were removed from males of the four mouse species and placed in 1,000 μl of M2 medium, and adipose tissue and blood vessels were removed. The clean structures were placed in a 500 μl drop of Human Tubular Fluid (HTF) medium (BSA supplemented) covered with mineral oil (Sigma-Aldrich, St. Louis, USA), from which spermatozoa were collected. Concentrations were determined with a Bürker hemocytometer. Spermatozoa were incubated in HTF for 30 min at 37°C with 5% CO_2_ in air for capacitation.

Fourteen hours after hCG injection, females were sacrificed by cervical dislocation and their oviducts were removed. COCs were obtained from the ampulla of the uterine tube and using a wide-bore pipette tip, placed in 500 μl of HTF medium. Each sample was inseminated with a final concentration of 1 × 10^6^ spermatozoa/ml, and 30 min after, each well was observed under an inverted microscope to assess sperm-oocyte binding. Gametes were co-incubated for 5 h at 37°C under 5% CO_2_ in air, after which, remaining CCs and attached sperm were removed by washing in HTF medium with a fine pipette; oocytes were then washed three times in potassium simplex optimized medium (KSOMaa) and placed in culture drop for 24 h at 37°C under 5% CO_2_ in air. Nine hours after insemination, the extent of successful fertilization was assessed in a group of presumptive zygotes by pronucleus visualization under a microscope. Another group of presumptive zygotes was confirmed 24 h after, by analyzing 2-cell stage embryos.

### Pronucleus Visualization

Nine hours after insemination, oocytes were incubated in a 100 μl drop of 10 pg Hoechst-33342 dye (bisbenzimide trihydrochloride, Sigma, Madrid, Spain), before being placed on a coverslip for viewing by a fluorescent microscope (Nikon Optiphot-2). The DNA + H-33342 complex was excited with UV at 355 nm light and epifluorescence emission at 465, and photographed. For this, a G 365 excitation filter, an FT 395 dichromatic beam splitter, and an LP 420 barrier filter were used. Both epifluorescent and brightfield photographs were taken using a Coolpix MDC Lens, Nikon, Japan.

### Molecular Analysis

#### Samples and Genomic DNA Isolation

A total of 23 species of the subfamily Murinae were included in this study (Table 1). DNA was extracted from ethanol-preserved tissues obtained from the collection of Preserved Mammalian Tissues of the “Institut des Sciences de l'Evolution of Montpellier” and from mice of the “Conservatoire Génétique de Souris Sauvages de Montpellier” (Montpellier, France). Total DNA was extracted using a QIAamp DNA Mini Kit (Qiagen, Hilden, Germany) following the manufacturer's recommendations.

#### Obtaining Ovarian RNA and cDNA Synthesis

Total RNA was isolated from ovaries of three species: *Mus mattheyi, Mus pahari*, and *Mastomys coucha* using RNAqueous® kit (Ambion, Austin Texas, USA) according to the manufacturer's instructions. The first-strand cDNA was synthesized with the SuperScript First-Strand Synthesis System kit for RT-PCR (Invitrogen-Life Technologies, Carlsbad, USA), according to the manufacturer's protocol.

#### Amplification and Sequencing of ZP Genes

PCR amplifications were made using genomic DNA or ovarian cDNA as templates. Primers were designed from *Mus musculus Zp1* (NM_009580), *Zp2* (NM_011775), and *Zp3* (NM_011776) sequences; in the case of *Zp4*, primers were designed from conserved regions of *Zp4* in *Mus musculus* (NR_027813) and *Rattus norvegicus* (NM_172330) (Supplementary Table 1). Overlapping fragments were analyzed to sequence the region of genomic DNA encompassing the exons 1–9 of the *Zp4* genes, which is 4,513 bp long for *Rattus norvegicus*. PCR amplification for DNA amplification was carried out in 50 μl reaction volume containing 5 μl of DNA or 2 μl of DNA, 0.5 μM of each primer, 200 μM of each dNTP, 2 mM MgCl_2_, and 1 IU of Taq DNA Polymerase (Fermentas, Waltham, USA) or 2.5 U of polymerase AmpliTaq Gold (Applied Biosystems, California, USA). PCR was carried out using a Mastercycler personal thermocycler (Eppendorf, Hamburg, Germany) or a T300 thermocycler (Biometra, Germany) following an initial denaturation cycle of 3 min at 95°C, and then 30 cycles of 1 min at 95°C, followed by 1 min at annealing temperature (depending on the primers) and then 1 min at 72°C. The final extension time was 10 min at 72°C. PCR products were analyzed by electrophoresis on 1.5% agarose gels. Four microliters of the PCR reaction mixture were mixed with loading buffer (Fermentas, Waltham, USA) and separated for 60 min at 90 V before visualizing under UV light using ethidium bromide (Sigma-Aldrich, St. Louis, USA).

Amplicons were carefully excised from the agarose gels and purified with the QIAquick Gel Extraction Kit Protocol (Qiagen) or DNA gel extraction kit (Millipore) or directly purified with the DNA Clean and Concentrator TM-5 (Zymo) according to the manufacturer's instructions. Amplicons were automatically sequenced using a 3,500 Genetic Analyzer (Applied Biosystems, California, USA) or send to Genome Express (Meylan, France). The new sequences were submitted to GenBank under the accession numbers: MH822867, MH822868, and MH822871 for *Mus mattheyi, Mus pahari*, and *Mastomys coucha*, respectively, (mRNA) and to EMBL under the accession numbers: LR990796-LR990832 for the DNA sequences.

### Bioinformatic Analysis

Sequences were analyzed to determine the degree of homology with other known sequences using the “BLAST program” (Basic Local Alignment Search Tool) (http://www.ncbi.nlm.nih.gov/blast/). Multiple sequence alignment was carried out using “Clustal Omega” (http://www.ebi.ac.uk/Tools/msa/clustalo/).

The amino acid sequences were analyzed using the software packages “signalP” (www.cbs.dtu.dk/services/SignalP/), “smart genome” (http://smart.embl-heidelberg.de/) to predict the signal peptide and different domains and “NetOGlyc” (www.cbs.dtu.dk/services/NetOGlyc) and “NetNglyc” (www.cbs.dtu.dk/services/NetNGlyc) to predict potential O-linked and N-linked glycosylation sites, respectively. The theoretical protein molecular weight and mature protein molecular weight were calculated with “PeptideMass” from “ExPASy” (http://web.expasy.org/peptide_mass/).

### Phylogenetic Analysis

To complete the dataset, ZP4 sequences from different muroid rodents (Table 1) were retrieved from GenBank and Ensembl gene predictions.

All these predictions were checked manually to detect annotation errors especially close to splicing sites. Similarity searches were performed using BLAST and BLAT against assembled genomes in Ensembl followed by a manual compilation of data to predict further genes or exons missing from the Ensembl predictions. It was also checked that the new sequences corresponded to a syntenic region of the corresponding chromosome. Only the exonic portions were kept for the phylogenetic analysis. Translated sequences were aligned using Muscle in Seaview (Gouy et al., 2010). The pseudogene sequences were added afterwards to the nucleotide alignment and manually aligned. The best-fit model of evolution (SYM+G) was determined using the Akaike information criterion (AIC; Akaike 1973), as implemented in jModelTest v2.1.7 (Darriba et al., 2012). Phylogenetic trees were reconstructed using two probabilistic approaches: maximum likelihood (ML) and Bayesian inferences (BI). The ML phylogeny was reconstructed with PhyML (Guindon et al., 2010). The robustness of each node was assessed with 1,000 bootstrap replicates. BI was performed using MrBayes v3.2 (Ronquist et al., 2012). Four independent runs of 10,000,000 generations sampled every 500th generation were performed. A burn-in period was determined graphically using Tracer1.7 (Rambaut et al., 2018). It was also checked that the effective sample sizes (ESSs) were above 200 and that the average SD of split frequencies remained <0.05 after the burn-in threshold. We discarded 10% of the trees and visualized the resulting tree with FigTree v1.4 (Rambaut, 2016). The robustness of the nodes was estimated with Posterior Probabilities (PP).

### Test for Evidence of Positive Selection

Selection analyses were made with the muroid datasets, modified to remove the pseudogene sequences, leading to the first alignment of 28 taxa with 711 bp (237 codons) and a second alignment with only the complete Zp4 sequences including 13 taxa (1,644 bp). The analyses were performed with CODEML from PAML4 (Yang, 2007). Data were analyzed under different models: M1a (neutral model), M2a (selection), M7 (beta distribution), and M8 (beta distribution and selection). The likelihood ratio test (LRT) of positive selection was performed on two pairs of models, M1a with M2a, and M7 with M8 (Yang, 2007).

### Proteomic Analysis

The expression of ZP proteins was studied using proteomic analysis in *Mastomys coucha, Mus mattheyi*, and *Mus pahari* ZP. Ovaries were trimmed using small scissors and dissected to remove fat and connective tissue. The solubilized ZP was obtained according to the protocol previously described by our group (Izquierdo-Rico et al., 2009; Jiménez-Movilla et al., 2009). Solubilized ZP was also obtained from oocytes, for which oocyte ZP was solubilized at 65°C in PBS buffer for 30 min. The analysis was carried out on an HPLC-MS system consisting of an Agilent 1100 Series HPLC (Agilent Technologies, Santa Clara, CA) equipped with a μ-well-plate autosampler and a capillary pump, and connected to an Agilent Ion-Trap XCT Plus mass spectrometer (Agilent Technologies, Santa Clara, CA) equipped with an electrospray (ESI) interface.

## Data Availability Statement

The datasets presented in this study can be found in online repositories. The names of the repository/repositories and accession number(s) can be found in the article/Supplementary Material.

## Ethics Statement

All animal procedures were performed following the Spanish Animal Protection Regulation, RD 1201/2005 which conforms to European Union Regulation 2003/65. All animal experiments were approved by the institutional review board of the University of Murcia according to the guide for Care and Use of Laboratory Animals as adopted by the Society for the Study of Reproduction.

## Author Contributions

MJI-R, CM-N, and PC performed molecular analysis and performed the bioinformatic analysis. MP-C, RL-B, and AG-A performed the *in vitro* fertilization analysis. PC and MA designed the study and conceived the project. MJI-R, CM-N, PC, and MA wrote the paper. All authors discussed the results and commented on the manuscript.

## Conflict of Interest

The authors declare that the research was conducted in the absence of any commercial or financial relationships that could be construed as a potential conflict of interest.

## References

[B1] Aghová T. Kimura Y. Bryja J. Dobigny G. Granjon L. Kergoat G. J. (2018). Fossils know it best: using a new set of fossil calibrations to improve the temporal phylogenetic framework of murid rodents (*Rodentia: Muridae*). Mol. Phylogenet. Evol. 128, 98–111. 10.1016/j.ympev.2018.07.017 30030180

[B2] Auffray J. Britton-Davidian J. (2012). “The house mouse and its relatives,” in Evolution of the House Mouse Cambridge Studies in Morphology and Molecules: New Paradigms in Evolutionary Biology, eds Macholán J. P. M. Baird S. Munclinger P. (Cambridge: Cambridge University Press), 1–34. 10.1017/CBO9781139044547.003

[B3] Avella M. A. Baibakov B. Dean J. (2014). A single domain of the ZP2 zona pellucida protein mediates gamete recognition in mice and humans. J. Cell Biol. 205, 801–809. 10.1083/jcb.201404025 24934154 PMC4068139

[B4] Avella M. A. Baibakov B. A. Jimenez-Movilla M. Sadusky A. B. Dean J. (2016). ZP2 peptide beads select human sperm *in vitro*, decoy mouse sperm *in vivo*, and provide reversible contraception. Sci. Transl. Med. 8:336ra60. 10.1016/j.fertnstert.2016.07.336 27122613 PMC6460476

[B5] Bae H. S. Jin Y. K. Ham S. Kim H. K. Shin H. Cho G. (2020). CRISRP/Cas9-mediated knockout of Mct8 reveals a functional involvement of Mct8 in testis and sperm development in a rat. Sci. Rep. 10:11148 10.1038/s41598-020-76579-0 32636400 PMC7341756

[B6] Baibakov B. Boggs N. A. Yauger B. Baibakov G. Dean J. (2012). Human sperm bind to the N-terminal domain of ZP2 in humanized zonae pellucidae in transgenic mice. J. Cell Biol. 197, 897–905. 10.1083/jcb.201203062 22734000 PMC3384420

[B7] Bausek N. Waclawek M. Schneider W. J. Wohlrab F. (2000). The major chicken egg envelope protein ZP1 is different from ZPB and is synthesized in the liver. J. Biol. Chem. 275, 28866–28872. 10.1074/jbc.275.37.28866 10979984

[B8] Bendtsen J. D. Nielsen H. von Heijne G. Brunak S. (2004). Improved prediction of signal peptides: SignalP 3.0. J. Mol. Biol. 340, 783–795. 10.1016/J.JMB.2004.05.028 15223320

[B9] Bleil J. D. Wassarman P. M. (1980). Structure and function of the zona pellucida: identification and characterization of the proteins of the mouse oocyte's zona pellucida. Dev. Biol. 76, 185–202. 10.1016/0012-1606(80)90371-1 7380091

[B10] Boja E. S. Hoodbhoy T. Fales H. M. Dean J. (2003). Structural characterization of native mouse zona pellucida proteins using mass spectrometry. J. Biol. Chem. 278, 34189–34202. 10.1074/jbc.M304026200 12799386

[B11] Boja E. S. Hoodbhoy T. Garfield M. Fales H. M. (2005). Structural conservation of mouse and rat zona pellucida glycoproteins. Probing the native rat zona pellucida proteome by mass spectrometry. Biochemistry 44, 16445–16460. 10.1021/bi051883f 16342937

[B12] Bork P. (1993). A trefoil domain in the major rabbit zona pellucida protein. Protein Sci. 2, 669–670. 10.1002/pro.5560020417 8518738 PMC2142363

[B13] Burkart A. D. Xiong B. Baibakov B. Jiménez-Movilla M. Dean J. (2012). Ovastacin, a cortical granule protease, cleaves ZP2 in the zona pellucida to prevent polyspermy. J. Cell Biol. 197, 37–44. 10.1083/jcb.201112094 22472438 PMC3317803

[B14] Callebaut I. Mornon J. P. Monget P. (2007). Isolated ZP-N domains constitute the N-terminal extensions of Zona Pellucida proteins. Bioinformatics 23, 1871–1874. 10.1093/bioinformatics/btm265 17510169

[B15] Cañestro C. Albalat R. Irimia M. Garcia-Fernàndez J. (2013). Impact of gene gains, losses and duplication modes on the origin and diversification of vertebrates. Semin. Cell Dev. Biol. 24, 83–94. 10.1016/j.semcdb.2012.12.008 23291262

[B16] Chevret P. Veyrunes F. Britton-Davidian J. (2005). Molecular phylogeny of the genus *Mus* (Rodentia: Murinae) based on mitochondrial and nuclear data. Biol. J. Linn. Soc. 84, 417–427. 10.1111/j.1095-8312.2005.00444.x

[B17] Darriba D. Taboada G. L. Doallo R. Posada D. (2012). jModelTest 2: more models, new heuristics and parallel computing. Nat. Methods 9:772. 10.1038/nmeth.2109 22847109 PMC4594756

[B18] Dean J. (2004). Reassessing the molecular biology of sperm-egg recognition with mouse genetics. Bioessays 26, 29–38. 10.1002/bies.10412 14696038

[B19] Dean M. D. Nachman M. W. (2009). Faster fertilization rate in conspecific versus heterospecific matings in house mice. Evolution 63, 20–28. 10.1111/j.1558-5646.2008.00499.x 18752610 PMC2908291

[B20] Dean J. (2007). The enigma of sperm-egg recognition in mice. Soc. Reprod. Fertil. Suppl. 63, 359–365. 17566284

[B21] Dorus S. Evans P. D. Wyckoff G. J. Sun S. C. Lahn B. T. (2004). Rate of molecular evolution of the seminal protein gene SEMG2 correlates with levels of female promiscuity. Nat. Genet. 36, 1326–1329. 10.1038/ng1471 15531881

[B22] Evsikov A. V. Graber J. H. Brockman J. M. Hampl A. Holbrook A. E. Singh P.. (2006). Cracking the egg: molecular dynamics and evolutionary aspects of the transition from the fully grown oocyte to embryo. Genes Dev. 20, 2713–2727. 10.1101/gad.1471006 17015433 PMC1578697

[B23] Fan Z. Li W. Lee S. R. Meng Q. Shi B. Bunch T. D.. (2014). Efficient gene targeting in golden Syrian hamsters by the CRISPR/Cas9 system. PLoS ONE 9:e0109755. 10.1371/journal.pone.0109755 25299451 PMC4192357

[B24] Feng J.-M. Tian H.-F. Hu Q.-M. Meng Y. Xiao H.-B. (2018). Evolution and multiple origins of zona pellucida genes in vertebrates. Biol. Open 7:bio.036137. 10.1242/bio.036137 30425109 PMC6262864

[B25] Frankenberg S. Renfree M. B. (2018). Conceptus coats of marsupials and monotremes. Curr. Top. Dev. Biol. 130, 357–377. 10.1016/bs.ctdb.2018.03.004 29853183

[B26] Ganguly A. Sharma R. K. Gupta S. K. (2008). Bonnet monkey (*Macaca radiata*) ovaries, like human oocytes, express four zona pellucida glycoproteins. Mol. Reprod. Dev. 75, 156–166. 10.1002/mrd.20808 17894386

[B27] Goudet G. Mugnier S. Callebaut I. Monget P. (2008). Phylogenetic analysis and identification of pseudogenes reveal a progressive loss of zona pellucida genes during evolution of vertebrates. Biol. Reprod. 78, 796–806. 10.1095/biolreprod.107.064568 18046012

[B28] Gouy M. Guindon S. Gascuel O. (2010). Sea view version 4: a multiplatform graphical user interface for sequence alignment and phylogenetic tree building. Mol. Biol. Evol. 27, 221–224. 10.1093/molbev/msp259 19854763

[B29] Greve J. M. Wassarman P. M. (1985). Mouse egg extracellular coat is a matrix of interconnected filaments possessing a structural repeat. J. Mol. Biol. 181, 253–264. 10.1016/0022-2836(85)90089-0 3845123

[B30] Guindon S. Dufayard J. F. Lefort V. Anisimova M. Hordijk W. Gascuel O. (2010). New algorithms and methods to estimate maximum-likelihood phylogenies: assessing the performance of PhyML 3.0. Syst. Biol. 59, 307–321. 10.1093/sysbio/syq010 20525638

[B31] Gupta S. K. (2018). The human egg's zona pellucida. Curr. Top. Dev. Biol. 130, 379–411. 10.1016/bs.ctdb.2018.01.001 29853184

[B32] Gupta S. K. Bhandari B. (2011). Acrosome reaction: relevance of zona pellucida glycoproteins. Asian J. Androl. 13, 97–105. 10.1038/aja.2010.72 21042299 PMC3739397

[B33] Gupta S. K. Bhandari B. Shrestha A. Biswal B. K. Palaniappan C. Malhotra S. S.. (2012). Mammalian zona pellucida glycoproteins: structure and function during fertilization. Cell Tissue Res. 349, 665–678. 10.1007/s00441-011-1319-y 22298023

[B34] Hedrick J. L. Wardrip N. J. (1987). On the macromolecular composition of the zona pellucida from porcine oocytes. Dev. Biol. 121, 478–488. 10.1016/0012-1606(87)90184-9 3556269

[B35] Hoodbhoy T. Joshi S. Boja E. S. Williams S. A. Stanley P. Dean J. (2005). Human sperm do not bind to rat zonae pellucidae despite the presence of four homologous glycoproteins. J. Biol. Chem. 280, 12721–12731. 10.1074/jbc.M413569200 15677449

[B36] Hourcade J. D. Pérez-Crespo M. Fernández-González R. Pintado B. Gutiérrez-Adán A. (2010). Selection against spermatozoa with fragmented DNA after postovulatory mating depends on the type of damage. Reprod. Biol. Endocrinol. 8:9. 10.1186/1477-7827-8-9 20113521 PMC2825232

[B37] Huang H.-L. Lv C. Zhao Y.-C. Li W. He X.-M. Li P.. (2014). Mutant ZP1 in familial infertility. N. Engl. J. Med. 370, 1220–1226. 10.1056/NEJMoa1308851 24670168 PMC4076492

[B38] Hughes D. C. Barratt C. L. R. (1999). Identification of the true human orthologue of the mouse Zp1 gene: evidence for greater complexity in the mammalian zona pellucida? Biochim. Biophys. Acta Gene Struct. Expr. 1447, 303–306. 10.1016/S0167-4781(99)00181-5 10542331

[B39] Izquierdo-Rico M. J. Jiménez-Movilla M. Llop E. Pérez-Oliva A. B. Ballesta J. Gutiérrez-Gallego R.. (2009). Hamster zona pellucida is formed by four glycoproteins: ZP1, ZP2, ZP3, and ZP4. J. Proteome Res. 8, 926–941. 10.1021/pr800568x 19159282

[B40] Jacobs L. L. Flynn L. J. Downs W. R. (1989). “Neogene rodents of South Asia”, in Papers on Fossil Rodents in Honor of Albert Elmer Wood. No. 33, Science Series, eds Black M. R. Dawson C. C. (Los Angeles, CA: Natural History Museum of Los Angeles), 157–177.

[B41] Jacobs L. L. Flynn L. J. Downs W. R. Barry J. C. (1990). “Quo vadis, antemus? The siwalik muroid record” in European Neogene Mammal Chronology, NATO ASI Series (Series A: Life Sciences), Vol. 180, eds Lindsay P. Fahlbush E. H. Mein V. (Boston, MA: Springer), 573-586. 10.1007/978-1-4899-2513-8_34

[B42] Jaeger J.-J. Tong H. Denys C. (1986). Age de la divergence Mus-Rattus: comparaison des données paléontologiques et moléculaires. Acad. Sci. Paris 302, 917–922.

[B43] Jansa S. A. Barker F. K. Voss R. S. (2014). The early diversification history of didelphid marsupials: a window into South America's “Splendid Isolation”. Evolution 68, 684–695. 10.1111/evo.12290 24125654

[B44] Jiménez-Movilla M. Martínez-Alonso E. Castells M. T. Izquierdo-Rico M. J. Saavedra M. D. Gutiérrez-Gallego R.. (2009). Cytochemical and biochemical evidences for a complex tridimensional structure of the hamster zona pellucida. Histol. Histopathol. 24, 599–609. 10.14670/HH-24.599 19283668

[B45] Kiefer S. M. Saling P. (2002). Proteolytic processing of human zona pellucida proteins1. Biol. Reprod. 66, 407–414. 10.1095/biolreprod66.2.407 11804956

[B46] Killingbeck E. E. Swanson W. J. (2018). Egg coat proteins across metazoan evolution. Curr. Top. Dev. Biol. 130, 443–488. 10.1016/bs.ctdb.2018.03.005 29853187 PMC6028277

[B47] Kingan S. B. Tatar M. Rand D. M. (2003). Reduced polymorphism in the chimpanzee semen coagulating protein, semenogelin I. J. Mol. Evol. 57, 159–169. 10.1007/s00239-002-2463-0 14562960

[B48] Krogh A. Larsson B. Von Heijne G. Sonnhammer E. L. L. (2001). Predicting transmembrane protein topology with a hidden Markov model: application to complete genomes. J. Mol. Biol. 305, 567–580. 10.1006/jmbi.2000.4315 11152613

[B49] Kudo K. Yonezawa N. Katsumata T. Aoki H. Nakano M. (1998). Localization of carbohydrate chains of pig sperm ligand in the glycoprotein ZPB of egg zona pellucida. Eur. J. Biochem. 252, 492–499. 10.1046/j.1432-1327.1998.2520492.x 9546665

[B50] Lamas-Toranzo I. Fonseca Balvís N. Querejeta-Fernández A. Izquierdo-Rico M. J. González-Brusi L. Lorenzo P. L.. (2019). ZP4 confers structural properties to the zona pellucida essential for embryo development. Elife 8:e48904. 10.7554/eLife.48904.018 31635692 PMC6805156

[B51] Lecompte E. Aplin K. Denys C. Catzeflis F. Chades M. Chevret P. (2008). Phylogeny and biogeography of African Murinae based on mitochondrial and nuclear gene sequences, with a new tribal classification of the subfamily. BMC Evol. Biol. 8:199. 10.1186/1471-2148-8-199 18616808 PMC2490707

[B52] Lefièvre L. Conner S. J. Salpekar A. Olufowobi O. Ashton P. Pavlovic B.. (2004). Four zona pellucida glycoproteins are expressed in the human. Hum. Reprod. 19, 1580–1586. 10.1093/humrep/deh301 15142998

[B53] Liu C. Litscher E. S. Mortillo S. Sakai Y. Kinloch R. A. Stewart C. L.. (1996). Targeted disruption of the mZP3 gene results in production of eggs lacking a zona pellucida and infertility in female mice. Proc. Natl. Acad. Sci. U.S.A. 93, 5431–5436. 10.1073/pnas.93.11.5431 8643592 PMC39263

[B54] Martín-Coello J. Benavent-Corai J. Roldan E. R. S. Gomendio M. (2009). Sperm competition promotes asymmetries in reproductive barriers between closely related species. Evolution 63, 613–623. 10.1111/j.1558-5646.2008.00585.x 19087184

[B55] Meheretu Y. Šumbera R. Bryja J. (2015). Enigmatic Ethiopian endemic rodent *Muriculus imberbis* (Rüppell 1842) represents a separate lineage within genus *Mus*. Mammalia 79, 15–23. 10.1515/mammalia-2013-0119

[B56] Meredith R. W. Westerman M. Case J. A. Springer M. S. (2008). A phylogeny and timescale for marsupial evolution based on sequences for five nuclear genes. J. Mammal. Evol. 15, 1–36. 10.1007/s10914-007-9062-6

[B57] Monné M. Jovine L. (2011). A structural view of egg coat architecture and function in fertilization1. Biol. Reprod. 85, 661–669. 10.1095/biolreprod.111.092098 21715714

[B58] Moros-Nicolás C. Chevret P. Izquierdo-Rico M. J. Holt W. V. Esteban-Díaz D. López-Béjar M.. (2018a). Composition of marsupial zona pellucida: a molecular and phylogenetic approach. Reprod. Fertil. Dev. 30, 721–733. 10.1071/RD16519 29162213

[B59] Moros-Nicolás C. Fouchécourt S. Goudet G. Monget P. (2018b). Genes encoding mammalian oviductal proteins involved in fertilization are subjected to gene death and positive selection. J. Mol. Evol. 86, 655–667. 10.1007/s00239-018-9878-0 30456442 PMC6267676

[B60] Moros-Nicolás C. Leza A. Chevret P. Guillén-Martínez A. González-Brusi L. Boué F.. (2018c). Analysis of ZP1 gene reveals differences in zona pellucida composition in carnivores. Reprod. Fertil. Dev. 30, 272–285. 10.1071/RD17022 28679462

[B61] Mugnier S. Dell'Aquila M. E. Pelaez J. Douet C. Ambruosi B. de Santis T.. (2009). New insights into the mechanisms of fertilization: comparison of the fertilization steps, composition, and structure of the zona pellucida between horses and pigs. Biol. Reprod. 81, 856–870. 10.1095/biolreprod.109.077651 19587333

[B62] Musser G. G. Carleton M. D. (2005). “Superfamily Muroidea,” in Mammal Species of the World: A Taxonomic and Geographic Reference, eds Wilson D. E. Reeder D. M. (Baltimore, MD: Johns Hopkins University), 894–1531.

[B63] Nishimura K. Dioguardi E. Nishio S. Villa A. Han L. Matsuda T.. (2019). Molecular basis of egg coat cross-linking sheds light on ZP1-associated female infertility. Nat. Commun. 10:3086. 10.1038/s41467-019-10931-5 31300655 PMC6626044

[B64] Noguchi S. Yonezawa N. Katsumata T. Hashizume K. Kuwayama M. Hamano S.. (1994). Characterization of the zona pellucida glycoproteins from bovine ovarian and fertilized eggs. Biochim. Biophys. Acta 1201, 7–14. 10.1016/0304-4165(94)90143-0 7918585

[B65] Nyakatura K. Bininda-Emonds O. R. P. (2012). Updating the evolutionary history of Carnivora (*Mammalia*): a new species-level supertree complete with divergence time estimates. BMC Biol. 10:12. 10.1186/1741-7007-10-12 22369503 PMC3307490

[B66] Pagès M. Chevret P. Gros-Balthazard M. Hughes S. Alcover J. A. Hutterer R.. (2012). Paleogenetic analyses reveal unsuspected phylogenetic affinities between mice and the extinct Malpaisomys insularis, an endemic rodent of the Canaries. PLoS ONE 7:e31123. 10.1371/journal.pone.0031123 22363563 PMC3283599

[B67] Rambaut A. Drummond A. J. Xie D. Baele G. Suchard M. A. (2018). Posterior summarization in bayesian phylogenetics using tracer 1.7. Syst. Biol. 67, 901–904. 10.1093/sysbio/syy032 29718447 PMC6101584

[B68] Rambaut A. (2016). FigTree. Available online at: https://github.com/rambaut/figtree/releases

[B69] Rankin T. Familari M. Lee E. Ginsberg A. Dwyer N. Blanchette-Mackie J.. (1996). Mice homozygous for an insertional mutation in the Zp3 gene lack a zona pellucida and are infertile. Development 122, 2903–2910. 8787763 10.1242/dev.122.9.2903

[B70] Rankin T. Talbot P. Lee E. Dean J. (1999). Abnormal zonae pellucidae in mice lacking ZP1 result in early embryonic loss. Development 126, 3847–3855. 10433913 10.1242/dev.126.17.3847

[B71] Rankin T. L. O'Brien M. Lee E. Wigglesworth K. Eppig J. Dean J. (2001). Defective zonae pellucidae in Zp2-null mice disrupt folliculogenesis, fertility and development. Development 128, 1119–1126. 11245577 10.1242/dev.128.7.1119

[B72] Roldan E. R. Yanagimachi R. (1989). Cross-fertilization between Syrian and Chinese hamsters. J. Exp. Zool. 250, 321–328. 10.1002/jez.1402500312 2760577

[B73] Roldan E. R. S. Vitullo A. D. Merani M. S. Von Lawzewitsch I. (1985). Cross fertilization *in vivo* and *in vitro* between three species of vesper mice, *Calomys* (Rodentia, Cricetidae). J. Exp. Zool. 233, 433–442. 10.1002/jez.1402330312 3882881

[B74] Ronquist F. Teslenko M. van der Mark P. Ayres D. Darling A. Höhna S.. (2012). MrBayes 3.2: efficient bayesian phylogenetic inference and model choice across a large model space. Syst. Biol. 61, 539–542. 10.1093/sysbio/sys029 22357727 PMC3329765

[B75] Shimada T. Aplin K. P. Suzuki H. (2010). *Mus lepidoides* (Muridae, Rodentia) of Central burma is a distinct species of potentially great evolutionary and biogeographic significance. Zool. Sci. 27, 449–459. 10.2108/zsj.27.449 20443693

[B76] Shu L. Suter M. J.-F. Räsänen K. (2015). Evolution of egg coats: linking molecular biology and ecology. Mol. Ecol. 24, 4052–4073. 10.1111/mec.13283 26096364

[B77] Spargo S. C. Hope R. M. (2003). Evolution and nomenclature of the zona pellucida gene family. Biol. Reprod. 68, 358–362. 10.1095/biolreprod.102.008086 12533396

[B78] Stetson I. Avilés M. Moros C. García-Vázquez F. A. Gimeno L. Torrecillas A.. (2015). Four glycoproteins are expressed in the cat zona pellucida. Theriogenology 83, 1162–1173. 10.1016/j.theriogenology.2014.12.019 25623231

[B79] Stetson I. Izquierdo-Rico M. J. Moros C. Chevret P. Lorenzo P. L. Ballesta J.. (2012). Rabbit zona pellucida composition: a molecular, proteomic and phylogenetic approach. J. Proteomics 75, 5920–5935. 10.1016/j.jprot.2012.07.027 22842159

[B80] Stsiapanava A. Xu C. Brunati M. Zamora-Caballero S. Schaeffer C. Bokhove M.. (2020). Cryo-EM structure of native human uromodulin, a zona pellucida module polymer. EMBO J. 39:e106807. 10.15252/embj.2020106807 33196145 PMC7737619

[B81] Sun L. Fang X. Chen Z. Zhang H. Zhang Z. Zhou P.. (2019). Compound heterozygous ZP1 mutations cause empty follicle syndrome in infertile sisters. Hum. Mutat. 40, 2001–2006. 10.1002/humu.23864 31292994

[B82] Suzuki H. Aplin K. (2012). “Phylogeny and biogeography of the genus *Mus* in Eurasia.,” in Evolution of the House Mouse (Cambridge Studies in Morphology and Molecules: New Paradigms in Evolutionary Biology, eds Macholán J. P. Baird S. Munclinger P. (Cambridge: Cambridge University Press), 35–64. 10.1017/CBO9781139044547.004

[B83] Swanson W. J. Nielsen R. Yang Q. (2003). Pervasive adaptive evolution in mammalian fertilization proteins. Mol. Biol. Evol. 20, 18–20. 10.1093/oxfordjournals.molbev.a004233 12519901

[B84] Swanson W. J. Yang Z. Wolfner M. F. Aquadro C. F. (2001). Positive Darwinian selection drives the evolution of several female reproductive proteins in mammals. Proc. Natl. Acad. Sci. U. S. A. 98, 2509–2514. 10.1073/pnas.051605998 11226269 PMC30168

[B85] Tanihara F. Nakai M. Kaneko H. Noguchi J. Otoi T. Kikuchi K. (2013). Evaluation of zona pellucida function for sperm penetration during *in vitro* fertilization in pigs. J. Reprod. Dev. 59, 385–392. 10.1262/jrd.2013-021 23666494 PMC3944356

[B86] Tian X. Pascal G. Fouchécourt S. Pontarotti P. Monget P. (2009). Gene birth, death, and divergence: the different scenarios of reproduction-related gene evolution. Biol. Reprod. 80, 616–621. 10.1095/biolreprod.108.073684 19129511

[B87] Torgerson D. G. Kulathinal R. J. Singh R. S. (2002). Mammalian sperm proteins are rapidly evolving: evidence of positive selection in functionally diverse genes. Mol. Biol. Evol. 19, 1973–1980. 10.1093/oxfordjournals.molbev.a004021 12411606

[B88] Veyrunes F. Dobigny G. Yang F. O'Brien P. C. M. Catalan J. Robinson T. J.. (2006). Phylogenomics of the genus *Mus* (Rodentia; Muridae): extensive genome repatterning is not restricted to the house mouse. Proc. R. Soc. B Biol. Sci. 273, 2925–2934. 10.1098/rspb.2006.3670 17015352 PMC1639516

[B89] Wassarman P. M. (1988). Zona pellucida glycoproteins. Annu. Rev. Biochem. 57, 415–442. 10.1146/annurev.bi.57.070188.002215 3052278

[B90] Wassarman P. M. Litscher E. S. (2009). The multifunctional zona pellucida and mammalian fertilization. J. Reprod. Immunol. 83, 45–49. 10.1016/j.jri.2009.06.259 19875177

[B91] Wu T. Cheng Y. Liu Z. Tao W. Zheng S. Wang D. (2018). Bioinformatic analyses of zona pellucida genes in vertebrates and their expression in Nile tilapia. Fish Physiol. Biochem. 44, 435–449. 10.1007/s10695-017-0434-4 29307115

[B92] Yanagimachi R. (1994). “Mammalian fertilization,” in Physiology of Reproduction, eds Knobily E. Neil E. (New York, NY: Raven Press), 189–317.

[B93] Yang Z. (2007). PAML 4: phylogenetic analysis by maximum likelihood. Mol. Biol. Evol. 24, 1586–1591. 10.1093/molbev/msm088 17483113

[B94] Yauger B. Boggs N. A. Dean J. (2011). Human ZP4 is not sufficient for taxon-specific sperm recognition of the zona pellucida in transgenic mice. Reproduction 141, 313–319. 10.1530/REP-10-0241 21173071 PMC3272266

[B95] Yuan P. Li R. Li D. Zheng L. Ou S. Zhao H.. (2019). Novel mutation in the ZP1 gene and clinical implications. J. Assist. Reprod. Genet. 36, 741–747. 10.1007/s10815-019-01404-1 30778819 PMC6505010

[B96] Zhang G. Cowled C. Shi Z. Huang Z. Bishop-Lilly K. A. Fang X.. (2013). Comparative analysis of bat genomes. Science 339, 456–460. 10.1126/science.1230835 23258410 PMC8782153

[B97] Zurano J. P. Magalhães F. M. Asato A. E. Silva G. Bidau C. J. Mesquita D. O.. (2019). Cetartiodactyla: updating a time-calibrated molecular phylogeny. Mol. Phylogenet. Evol. 133, 256–262. 10.1016/j.ympev.2018.12.015 30562611

