# Supplementary material for: ZP4 Is Present in Murine Zona Pellucida and Is Not Responsible for the Specific Gamete Interaction
Source: Front Cell Dev Biol. 2021 Jan 18;8:626679. doi: 10.3389/fcell.2020.626679 (PMC7848090; doi:10.3389/fcell.2020.626679)
Supplement: Supplementary file 1 [file Table_1.docx]

**Table S1.** Primers used for the amplification and sequencing of *Mus mattheyi, Mus pahari* and *Mastomys coucha* ZPs mRNA and murine *Zp4* genomic DNA.

| **ZP** | **Pb (f/r)** | **Sequence (5’- 3’)** | **Tm (ºC)** |
| --- | --- | --- | --- |
| ***Zp1* mRNA** | | | |
| ZP1 | 19 (f) | ATCATGGCCTGGGGTTGTT | 58 |
| ZP1 | 20 (r) | TCCATGTGTCAAGGCTGTTT | 58 |
| ZP1 | 20 (f) | AGAAGACAAGTGCTTTTGTG | 56 |
| ZP1 | 18 (r) | GGCCCAGATCAGACCCAC | 60 |
| ZP1 | 20 (f) | GTGGCTACTTTACCTTGGTC | 60 |
| ZP1 | 20 (r) | TAGATGAGTTGTTCGCCGAC | 60 |
| ZP1 | 19 (f) | GACATTGTGAGCTCTCCAG | 58 |
| ZP1 | 21 (r) | TTTAATATCTGATGCCTTCCC | 58 |
| ZP1 | 19 (f) | ACTCCAGCTCAAGAATGCT | 56 |
| ZP1 | 21 (r) | AGTCATAGCTGTATTCAAAGC | 58 |
| ***Zp2* mRNA** | | | |
| ZP2 | 20 (f) | CTTTGAGGTCTACAGCCACC | 62 |
| ZP2 | 20 (r) | TGTAAGGCCGTTGGTAGGAT | 60 |
| ZP2 | 20 (f) | GCCAGCCAATCTACATGGAA | 60 |
| ZP2 | 20 (r) | GGCAAGTCACAGAGCACAGA | 62 |
| ***Zp3* mRNA** | | | |
| ZP3 | 19 (f) | ACCTCACCCTTGGCTCAGA | 60 |
| ZP3 | 19 (r) | ACCTCACCCTTGGCTCAGA | 58 |
| ZP3 | 21 (f) | TACATCACCTGCCATCTCAAA | 60 |
| ZP3 | 19 (r) | CAGGGTCAGGAATGCCACT | 60 |
| ***Zp4* mRNA** | | | |
| ZP4 | 22 (f) | GCTAGGCTAGTGAAGGAGAAAG | 66 |
| ZP4 | 23 (r) | TGCAGTCAGTTTTATTGAGACTC | 64 |
| ZP4 | 20 (f) | GCTTAAGTGTCCTTTGGATC | 58 |
| ZP4 | 19 (r) | GAGCCCCAATGCTTTCTAC | 58 |
| ZP4 | 21 (r) | GGGATTCATCTCAATTTTCTG | 58 |
| ZP4 | 20 (f) | TATCAGACCAAAAGGATCCC | 58 |
| ZP4 | 23 (r) | NNNATTCATCTCAATTTTCTGAT* | 56-62 |
| ZP4 | 21 (r) | NNNTCATCTCWRTTTYCTGRT* | 56-62 |
| ***ZP4* genomic DNA** | | | |
| ZP4 | 18 (f) | CCACCTTTGTCCTGTTCC | 56 |
| ZP4 | 18 (r) | GGAACAGGACAAAGGTGG | 56 |
| ZP4 | 18 (f) | GGTACATGGGTGATGGAC | 56 |
| ZP4 | 20 (f) | GGTCTGAGAACCCTTTAATC | 58 |
| ZP4 | 18 (f) | GATCCACCTGGCTCTGTC | 58 |
| ZP4 | 18 (r) | GGCTCTTCACATGCCTTC | 56 |
| ZP4 | 17 (r) | AAGCCAACGAGAGATCC | 52 |
| ZP4 | 19 (f) | CCAGGTGTGCTCCACTGTG | 62 |
| ZP4 | 21 (f) | GGGTAAGCTCTGCCTACTTGC | 66 |
| ZP4 | 21 (r) | GCAAGTAGGCAGAGCTTACCC | 66 |
| ZP4 | 21 (f) | CTCTAAGGAGTACTCTGTGGC | 64 |
| ZP4 | 23 (f) | GAGACTGCTTAAGTGCCCTTTGG | 70 |
| ZP4 | 20 (f) | TACAGATCACTGGAGACCAG | 60 |
| ZP4 | 21 (r) | CAAGAGCACCTTCTAGGCAAG | 64 |
| ZP4 | 19 (r) | GCACTCATGAAGCTGAAGG | 58 |
| ZP4 | 23 (r) | GTTGCTGTATCGGGTAATAGAGC | 68 |
| ZP4 | 22 (f) | GCTAGGCTAGTGAAGGAGAAAG | 66 |
| *N=A+C+G+T *W=A+T *R=A+G *Y=C+T | | | |
