# Supplementary material for: ZP4 Is Present in Murine Zona Pellucida and Is Not Responsible for the Specific Gamete Interaction
Source: Front Cell Dev Biol. 2021 Jan 18;8:626679. doi: 10.3389/fcell.2020.626679 (PMC7848090; doi:10.3389/fcell.2020.626679)
Supplement: Supplementary file 2 [file Table_2.pdf]

Table S2. Primers used for the amplification of *Mus mattheyi, Mus pahari* and *Mastomys coucha* ZPs mRNA showed in Figure 3.

Species	Primer name	Sequence
Mus mattheyi	ZP1-Fw	ATCATGGCCTGGGGTTGTT
	ZP1-Rv	TCCATGTGTCAAGGCTGTTT
	ZP2-Fw	GCCAGCCAATCTACATGGAA
	ZP2-Rv	GGCAAGTCACAGAGCACAGA
	ZP3-Fw	ACCTCACCCTTGGCTCAGA
	ZP3-Rv	CGAAGAGAGAAAGCCAGTTT
	ZP4-Fw	TATCAGACCAAAAGGATCCC
	ZP4-Rv	NNNATTCATCTCAATTTTCTGAT
Mus pahari	ZP1-Fw	AGAAGACAAGTGCTTTTGTG
	ZP1-Rv	TTTAATATCTGATGCCTTCCC
	ZP2-Fw	GCCAGCCAATCTACATGGAA
	ZP2-Rv	GGCAAGTCACAGAGCACAGA
	ZP3-Fw	TACATCACCTGCCATCTCAAA
	ZP3-Rv	CAGGGTCAGGAATGCCACT
	ZP4-Fw	TATCAGACCAAAAGGATCCC
	ZP4-Rv	NNNATTCATCTCAATTTTCTGAT
Mastomys coucha	ZP1-Fw	ATCATGGCCTGGGGTTGTT
	ZP1-Rv	TAGATGAGTTGTTCGCCGAC
	ZP2-Fw	GCCAGCCAATCTACATGGAA
	ZP2-Rv	GGCAAGTCACAGAGCACAGA
	ZP3-Fw	ACCTCACCCTTGGCTCAGA
	ZP3-Rv	CAGGGTCAGGAATGCCACT
	ZP4-Fw	TATCAGACCAAAAGGATCCC
	ZP4-Rv	NNNATTCATCTCAATTTTCTGAT
