# Supplementary material for: ZP4 Is Present in Murine Zona Pellucida and Is Not Responsible for the Specific Gamete Interaction
Source: Front Cell Dev Biol. 2021 Jan 18;8:626679. doi: 10.3389/fcell.2020.626679 (PMC7848090; doi:10.3389/fcell.2020.626679)
Supplement: Supplementary file 3 [file Image_1.pdf]

Fig. S1. Nucleotide and deduced amino acid sequence of a) *Mus mattheyi* and b) *Mus pahari* Zp1. The initial and final codons are in pink. The signal peptide is marked in green. Trefoil domain is shown in blue. The ZP module is shown in red. The consensus furin cleavage-site is underlined. The transmembrane domain is marked in orange. Sequences were submitted to GenBank with the following accession numbers MH822869.1 for *Mus mattheyi* and MH822870.1 for *Mus pahari*.

a)

```
   1 atggccgtgggtgttgtttgtgtggtccttctgctggcagcagctcctctacgattgggt
   1  M  A  V  G  V  V  C  V  V  L  L  L  A  A  A  P  L  R  L  G
  61 cagcgtctgcatctcgagcctggctttgaatacagctatgactgtggggtacggggcatg
  21  Q  R  L  H  L  E  P  G  F  E  Y  S  Y  D  C  G  V  R  G  M
 121 cagctgctggtgttccccaggccaaaccagactgtccaattcaaggtgctggatgaattt
  41  Q  L  L  V  F  P  R  P  N  Q  T  V  Q  F  K  V  L  D  E  F
 181 gggaaccggtttgaggtgaataactgctctatctgctaccactgggtgagcagtgaggcc
  61  G  N  R  F  E  V  N  N  C  S  I  C  Y  H  W  V  S  S  E  A
 241 caggagcgcacagtgttctcagctgaatataaaggctgccatgttctggagaaggatggg
  81  Q  E  R  T  V  F  S  A  E  Y  K  G  C  H  V  L  E  K  D  G
 301 cagttccacctgagagtgttcatacaagccgtcctacccaatggccgtgtggatgtagca
 101  Q  F  H  L  R  V  F  I  Q  A  V  L  P  N  G  R  V  D  V  A
 361 caagatgtcactctgatctgtcccaaaccagaccacatcatgactccagacccgtacctg
 121  Q  D  V  T  L  I  C  P  K  P  D  H  I  M  T  P  D  P  Y  L
 421 gctccacccaccacacctgagccttttacacctcatacttttgcccttcacctcatccct
 141  A  P  P  T  T  P  E  P  F  T  P  H  T  F  A  L  H  L  I  P
 481 ggccacaccttggctgggtctggccacactggccttactacgttgtacccagagcacagc
 161  G  H  T  L  A  G  S  G  H  T  G  L  T  T  L  Y  P  E  H  S
 541 ttcacccatccaactcctgctccaccatccccaggacctggacctgctggacccactgca
 181  F  T  H  P  T  P  A  P  P  S  P  G  P  G  P  A  G  P  T  A
 601 cctcactcccaatggggcactctggagccctgggaattgactgagctggattctgtgggt
 201  P  H  S  Q  W  G  T  L  E  P  W  E  L  T  E  L  D  S  V  G
 661 actcatctgccccaggagcagtgccaggtatcctccaggcacatcccatgcatggtaaaa
 221  T  H  L  P  Q  E  Q  C  Q  V  S  S  R  H  I  P  C  M  V  K
 721 ggaagtcctaaggaagcctgtcagcaggctggctgctgctatgacagtgccaaagaagag
 241  G  S  P  K  E  A  C  Q  Q  A  G  C  C  Y  D  S  A  K  E  E
 781 ccctgttactacggcaacacagtcactctccagtgtttcaaaagtggctactttaccttg
 261  P  C  Y  Y  G  N  T  V  T  L  Q  C  F  K  S  G  Y  F  T  L
 841 gtcatgtcgcaagaaacagccttgacacacggagtcatgctggacaatgtccacctggcc
 281  V  M  S  Q  E  T  A  L  T  H  G  V  M  L  D  N  V  H  L  A
 901 tatgcctccaatggatgcccccctacccagaagacaagtgcttttgtggtctttcgtgtc
 301  Y  A  S  N  G  C  P  P  T  Q  K  T  S  A  F  V  V  F  R  V
 961 ccttttaccctctgtggaacaacaatccaggtggtcggcgagcagcttatctatgagaac
 321  P  F  T  L  C  G  T  T  I  Q  V  V  G  E  Q  L  I  Y  E  N
1021 cagctggtgtctgacattgatgtccaaaaggggccacagggttccatcactcgggacagt
 341  Q  L  V  S  D  I  D  V  Q  K  G  P  Q  G  S  I  T  R  D  S
1081 gtcttccggcttcacgttcgctgcatcttcaatgctagtgatttcctgcccatccaggca
 361  V  F  R  L  H  V  R  C  I  F  N  A  S  D  F  L  P  I  Q  A
1141 tctatcttctcacctcaaccacctgcccccgtgactcagtctggaccccctgaggctggaa
 381  S  I  F  S  P  Q  P  P  A  P  V  T  Q  S  G  P  L  R  L  E
1201 ctgaggattgccacggataagactttcagctcctactatcaggggagtgactatcccctt
 401  L  R  I  A  T  D  K  T  F  S  S  Y  Y  Q  G  S  D  Y  P  L
1261 gtgagactgcttcaggagccagtctatgtagaggtccgactcctacagagaactgatccc
 421  V  R  L  L  Q  E  P  V  Y  V  E  V  R  L  L  Q  R  T  D  P
1321 agtctggttctggtgctacaccagtgctgggccacacccaccgccagccccattgagcag
 441  S  L  V  L  V  L  H  Q  C  W  A  T  P  T  A  S  P  I  E  Q
1381 ccccaatggcccattctgtcagatgggtgtcctttcaagggtgacaactacagaacacaa
```

```
 461  P  Q  W  P  I  L  S  D  G  C  P  F  K  G  D  N  Y  R  T  Q
1441 gtggtggccacagacaaggaggcgcttcccttctggtcccactatcagcgcttcaccatc
 481  V  V  A  T  D  K  E  A  L  P  F  W  S  H  Y  Q  R  F  T  I
1501 gccaccttcacactccttgacttcagttcccagaatgcccttaggggacaggtctatttc
 501  A  T  F  T  L  L  D  F  S  S  Q  N  A  L  R  G  Q  V  Y  F
1561 ttctgtagtgcctctgcctgccaccctgtggggtctgacacatgttctactacgtgtgac
 521  F  C  S  A  S  A  C  H  P  V  G  S  D  T  C  S  T  T  C  D
1621 tctgggatagcaaggcgtcgaagatcctctggtcaccacaacagcactctccgggccctg
 541  S  G  I  A  R  R  R  R  S  S  G  H  H  N  S  T  L  R  A  L
1681 gacattgtaagctctccaggggcagtgggctttgaggatactgctaaacttgagccctca
 561  D  I  V  S  S  P  G  A  V  G  F  E  D  T  A  K  L  E  P  S
1741 ggttccagcaggaactccagttcaagaatgctgctcctgctgctggccatcaccctagcc
 581  G  S  S  R  N  S  S  S  R  M  L  L  L  L  L  A  I  T  L  A
1801 ctggcggccggtatctttgtgggtctgatctgggcctgggcccagaagctctgggaaggc
 601  L  A  A  G  I  F  V  G  L  I  W  A  W  A  Q  K  L  W  E  G
1861 atcagatattaa
 621  I  R  Y  -
```

b)

```
   1 atgggctggggttgttttgtggccctgcttctgctggcggcagctccccctaagattggct
   1  M  G  W  G  C  F  V  A  L  L  L  L  A  A  A  P  L  R  L  A
  61 cagcatctgcatctggagcctggcttggaatacagctatgactgtggggtacggggaatg
  21  Q  H  L  H  L  E  P  G  L  E  Y  S  Y  D  C  G  V  R  G  M
 121 cagctgctggtattccccaggccaaaccagactatcgaatccaaggtgctggatgaattt
  41  Q  L  L  V  F  P  R  P  N  Q  T  I  E  S  K  V  L  D  E  F
 181 gggaaccggtttgaggtgaataactgctctatctgctaccactgggtgaccagtgaggcc
  61  G  N  R  F  E  V  N  N  C  S  I  C  Y  H  W  V  T  S  E  A
 241 cagaagcgcacagtattctcagctgattaccaaggctgccacgttctggagaaggatggg
  81  Q  K  R  T  V  F  S  A  D  Y  Q  G  C  H  V  L  E  K  D  G
 301 cggttccacctgagggtgttcatacaagccgtcctacccaatggccgtgtggatatagca
 101  R  F  H  L  R  V  F  I  Q  A  V  L  P  N  G  R  V  D  I  A
 361 cgagatgtcactctgatctgtcccaaaccagaccacatcatgactccggacccctacctg
 121  R  D  V  T  L  I  C  P  K  P  D  H  I  M  T  P  D  P  Y  L
 421 gctccacccaccacacctgagccttttacacctcctactttgcccttcactccatccct
 141  A  P  P  T  T  P  E  P  F  T  P  P  T  F  A  L  H  S  I  P
 481 ggccacaccttggctgggtctggccacactggtctcactacgttgtactcagagcacagc
 161  G  H  T  L  A  G  S  G  H  T  G  L  T  T  L  Y  S  E  H  S
 541 ttcacccatccaactcctgccccaccatccccaggacctggacctgctggacccaccgta
 181  F  T  H  P  T  P  A  P  P  S  P  G  P  G  P  A  G  P  T  V
 601 cctcactcccaatggggcacgttggagccctgggaattgactgagctggattctgtaggc
 201  P  H  S  Q  W  G  T  L  E  P  W  E  L  T  E  L  D  S  V  G
 661 acccatctgccccaggagcagtgccaggtagcctccgggcacatcccgtgcatggtaaaa
 221  T  H  L  P  Q  E  Q  C  Q  V  A  S  G  H  I  P  C  M  V  K
 721 ggaagttccaaggaagcctgtcagcaggctggctgctgctacgacagtaccaaagaagag
 241  G  S  S  K  E  A  C  Q  Q  A  G  C  C  Y  D  S  T  K  E  E
 781 ccctgttactatggcaacacagtcactctccagtgtttcaaaagtggctactttaccttg
 261  P  C  Y  Y  G  N  T  V  T  L  Q  C  F  K  S  G  Y  F  T  L
 841 gtcgtgtcacaagaaacagccttgacacatggagtcatgctggacaatgtccgtctggcc
 281  V  V  S  Q  E  T  A  L  T  H  G  V  M  L  D  N  V  R  L  A
 901 tatgcccccaacggatgccccctacccagaagacaagtgctttcgtggtcttccatgtc
 301  Y  A  P  N  G  C  P  P  T  Q  K  T  S  A  F  V  V  F  H  V
 961 cctctcaccctctgtggaacggcaatccaggtggttggtgagcagctcatctatgagaac
 321  P  L  T  L  C  G  T  A  I  Q  V  V  G  E  Q  L  I  Y  E  N
1021 cagctggtgtctgacattgatatccaaaagggcccacaaggttccatcactcgtgacagt
 341  Q  L  V  S  D  I  D  I  Q  K  G  P  Q  G  S  I  T  R  D  S
1081 gtcttccggcttcatgttcgctgcatcttcaatgctagcgatttcctgcccatccaggca
 361  V  F  R  L  H  V  R  C  I  F  N  A  S  D  F  L  P  I  Q  A
1141 tctatcctctcaccccaaccacctgcccccgtgactcagtctggacccctgaagctggaa
```

381 S I L S P Q P P A P V T Q S G P L K L E
1201 ctgaggattgccacggataagactttcagctcctactatcaggggagtgactatcccctt
401 L R I A T D K T F S S Y Y Q G S D Y P L
1261 gtgagactgctccaggaaccagtctacatagaggtccgactcctgcagagaactgatccc
421 V R L L Q E P V Y I E V R L L Q R T D P
1321 agtctggtgctggtgctacaccagtgctgggccacgcccaccaccagcccctttgagcag
441 S L V L V L H Q C W A T P T T S P F E Q
1381 ccccaatggcccattctgtcagatgggtgtcctttcaagggtgacaactacagaacacaa
461 P Q W P I L S D G C P F K G D N Y R T Q
1441 gtggtggctgcagacagggaggcgcttcccttctggtcccactatcagcggttcaccatt
481 V V A A D R E A L P F W S H Y Q R F T I
1501 gccaccttcacgctccttgactccagttcccagaatgccctaaggggacaggtctatttc
501 A T F T L L D S S S Q N A L R G Q V Y F
1561 ttctgtagtgcttctgcctgccaccctgtggggtccaacacatgctctactacatgtgac
521 F C S A S A C H P V G S N T C S T T C D
1621 tctgggatagcaaggcgtcgacgatcctctagtcaccacaacagcgttctccgggccctg
541 S G I A R R R R S S S H H N S V L R A L
1681 gacattgtgagctctccaggggcagtgggctttgaggatgctgctaaacttgagccctca
561 D I V S S P G A V G F E D A A K L E P S
1741 ggttccagcaggagctccagttcaagagtgctgctcccgctgctggccgtcaccctagcc
581 G S S R S S S S R V L L P L L A V T L A
1801 atggcggccggtatctttgtgggtctgatctgggcctgggcccagaaactctgggaaggc
601 M A A G I F V G L I W A W A Q K L W E G
1861 atcagatat**taa**
621 I R Y -
