# Supplementary material for: ZP4 Is Present in Murine Zona Pellucida and Is Not Responsible for the Specific Gamete Interaction
Source: Front Cell Dev Biol. 2021 Jan 18;8:626679. doi: 10.3389/fcell.2020.626679 (PMC7848090; doi:10.3389/fcell.2020.626679)
Supplement: Supplementary file 4 [file Image_2.pdf]

Fig. S2. Nucleotide and deduced amino acid sequence of a) *Mus mattheyi* and b) *Mus pahari* (b) Zp4. The initial and final codons are in pink. The signal peptide is marked in green. Trefoil domain is shown in blue. The ZP module is shown in red. The consensus furin cleavage-site is underlined. The transmembrane domain is marked in orange. Sequences were submitted to GenBank with the following accession numbers: MH822867.1 for *Mus mattheyi* and MH822868.1 for *Mus pahari*.

a)

```
   1 atggctggccaggctctaaggagcactttgtggcttctgccgagcatcttgctgtgtttc
   1  M  A  G  Q  A  L  R  S  T  L  W  L  L  P  S  I  L  L  C  F
  61 ccattctgtcctcccttgagtggccagcatgtgactgaattgccaggtgtgctccactgt
  21  P  F  C  P  P  L  S  G  Q  H  V  T  E  L  P  G  V  L  H  C
 121 gggttacagagcttccagtttactgcgaacctcagcctgggggcagagagtcctgtgcta
  41  G  L  Q  S  F  Q  F  T  A  N  L  S  L  G  A  E  S  P  V  L
 181 acagcttgggatagccaagggctgccacacaggcttaagaatgactctgactgtggtata
  61  T  A  W  D  S  Q  G  L  P  H  R  L  K  N  D  S  D  C  G  I
 241 tggatgacggacagtcctgatgggtttctggtattgaaagccacctacaatggctgctat
  81  W  M  T  D  S  P  D  G  F  L  V  L  K  A  T  Y  N  G  C  Y
 301 gtcactctgaagggctcccattatgtcatgatggtcagcatgcaagaggtagatgtagct
 101  V  T  L  K  G  S  H  Y  V  M  M  V  S  M  Q  E  V  D  V  A
 361 ggaaatatgacaggaacaagagagagactgcttaagtgccctttggatcttcgcgcccca
 121  G  N  M  T  G  T  R  E  R  L  L  K  C  P  L  D  L  R  A  P
 421 gatgcaccaagtgctgaagtgtgcagtcctgtgccagtaaaggaaaggctgccctgtgct
 141  D  A  P  S  A  E  V  C  S  P  V  P  V  K  E  R  L  P  C  A
 481 ccctcgcccatctccagaggagactgtgaagaggtgggctgctgctacagctctgaagaa
 161  P  S  P  I  S  R  G  D  C  E  E  V  G  C  C  Y  S  S  E  E
 541 gaagaggcgggttcctgttactatggaaacacagtgacctcccgttgtaccagggagggc
 181  E  E  A  G  S  C  Y  Y  G  N  T  V  T  S  R  C  T  R  E  G
 601 cgcttttccattgctgtgtccaggaatgcaacctcaccacccctgcgcttggattcccta
 201  R  F  S  I  A  V  S  R  N  A  T  S  P  P  L  R  L  D  S  L
 661 cacctggtcttcaggaacagcagtgggtgtgatcctgtgatgatgacatccacctttgtc
 221  H  L  V  F  R  N  S  S  G  C  D  P  V  M  M  T  S  T  F  V
 721 ctgttccaatttccatttacttcctgtgggaccgcacggaggatcactggagaccaggct
 241  L  F  Q  F  P  F  T  S  C  G  T  A  R  R  I  T  G  D  Q  A
 781 gtgtatgaaaatgagctagtggccattccggatgtgcaagcttggggcagaagctctatt
 261  V  Y  E  N  E  L  V  A  I  P  D  V  Q  A  W  G  R  S  S  I
 841 acccgagacagcaacttcaggctccgagtcagctgcacctactctgctctcagcaacaca
 281  T  R  D  S  N  F  R  L  R  V  S  C  T  Y  S  A  L  S  N  T
 901 tcccctattaacatgcaagtgctggctctcccaccaccccttcctaagacccagcctggg
 301  S  P  I  N  M  Q  V  L  A  L  P  P  P  L  P  K  T  Q  P  G
 961 cccctctctctggaacttcagattgccaaggataaaagctatggttcttactatggttct
 321  P  L  S  L  E  L  Q  I  A  K  D  K  S  Y  G  S  Y  Y  G  S
1021 gatgcctacccactggtaaaattcctccaggatcctatttatgtggaggtctccatcatt
 341  D  A  Y  P  L  V  K  F  L  Q  D  P  I  Y  V  E  V  S  I  I
1081 cacagaacagatccctcattgggtctgctgctagatcaatgttgggccacacctggctct
 361  H  R  T  D  P  S  L  G  L  L  L  D  Q  C  W  A  T  P  G  S
1141 aatccttttcatcaaccacagtggccaatcctggtgaagggatgcccatatgctggagac
 381  N  P  F  H  Q  P  Q  W  P  I  L  V  K  G  C  P  Y  A  G  D
1201 aactatcagaccaaaaggatccctgtccagaaaacatcaagtccctttccatctcatcac
```

```
 401  N  Y  Q  T  K  R  I  P  V  Q  K  T  S  S  P  F  P  S  H  H
1261 cagcgcttcagcatcgataccttcagcttcatgagtgctgtaagggagaagcagctttta
 421  Q  R  F  S  I  D  T  F  S  F  M  S  A  V  R  E  K  Q  L  L
1321 agtggacaggtgtacctgcactgcagtgcatcagtctgccagcctgctgggatgccatcc
 441  S  G  Q  V  Y  L  H  C  S  A  S  V  C  Q  P  A  G  M  P  S
1381 tgtgtgacagtctgtcctgcttccaggagaagaagaaaatctgagcttcattttgagacc
 461  C  V  T  V  C  P  A  S  R  R  R  R  K  S  E  L  H  F  E  T
1441 accaccagcatatctagcaaaggcccccctgatcctcctccaagccactaaggactctgca
 481  T  T  S  I  S  S  K  G  P  L  I  L  L  Q  A  T  K  D  S  A
1501 gacatgcttcatagacactcacgcacccctgtggattctactgctctgtgggtcatgggg
 501  D  M  L  H  R  H  S  R  T  P  V  D  S  T  A  L  W  V  M  G
1561 ctttctgcaactgtgatcatcactggggtctttgtagtatcctacttggccatcagaaaa
 521  L  S  A  T  V  I  I  T  G  V  F  V  V  S  Y  L  A  I  R  K
1621 ttgagatga
 541  L  R  -
```

b)

```
   1 atggctgggcaggctctaaggagcactctgtggcttctgccaagcatctttctgtgtttc
   1  M  A  G  Q  A  L  R  S  T  L  W  L  L  P  S  I  F  L  C  F
  61 ccgttctgtcctcccttgagtggccagcatgtgactgagttgccaggtgtgctccactgt
  21  P  F  C  P  P  L  S  G  Q  H  V  T  E  L  P  G  V  L  H  C
 121 aggttacagagcttccagtttactgtgaacctcagcctggaggcagagagtcctgtgcta
  41  R  L  Q  S  F  Q  F  T  V  N  L  S  L  E  A  E  S  P  V  L
 181 acagcttgggatagccaagggctgccacacaggcttaagaatgactctgactgtggtaca
  61  T  A  W  D  S  Q  G  L  P  H  R  L  K  N  D  S  D  C  G  T
 241 tgggtgatggacagtcctgatggattttggtattggaagccacctacaatggctgctat
  81  W  V  M  D  S  P  D  G  F  L  V  L  E  A  T  Y  N  G  C  Y
 301 gtcactctggagggctcccattatgtcatgatggtcggcgtgcaagaggtagatgtagcc
 101  V  T  L  E  G  S  H  Y  V  M  M  V  G  V  Q  E  V  D  V  A
 361 ggaaatataacgggaaggagagagagactgctcaagtgccctgtggatcttcacaccaca
 121  G  N  I  T  G  R  R  E  R  L  L  K  C  P  V  D  L  H  T  T
 421 gatgcatcaaatgctgaagtgtgcagtcctgtgccagtaaaggaaaggctgccctgtgct
 141  D  A  S  N  A  E  V  C  S  P  V  P  V  K  E  R  L  P  C  A
 481 ccctcgcccatctccagaggagattgtgaagaggcaggctgctgctacagctccgaagag
 161  P  S  P  I  S  R  G  D  C  E  E  A  G  C  C  Y  S  S  E  E
 541 gaagaggcaggttcctgttactatggaaacacagtgacctcccgttgcaccagggagggc
 181  E  E  A  G  S  C  Y  Y  G  N  T  V  T  S  R  C  T  R  E  G
 601 cgcttttccattgctgtgtccaggaatgcaacctcgccacccctgcacttggattcccta
 201  R  F  S  I  A  V  S  R  N  A  T  S  P  P  L  H  L  D  S  L
 661 cgcttggtcttcagggacaacagtgcgtgtgatcctgtgatgacaacagccacctttgtc
 221  R  L  V  F  R  D  N  S  A  C  D  P  V  M  T  T  A  T  F  V
 721 ctgttccaatttccatttacttcctgtgggaccacacggcggatcactggagacaaggcc
 241  L  F  Q  F  P  F  T  S  C  G  T  T  R  R  I  T  G  D  K  A
 781 gtgtatgaaaatgaactagtggccattcgggatgtgcaagcttggggcagaagctctatt
 261  V  Y  E  N  E  L  V  A  I  R  D  V  Q  A  W  G  R  S  S  I
 841 acccgagacagcaacttcaggctccgagtcagctgcatttactctgctctcagcaacaca
 281  T  R  D  S  N  F  R  L  R  V  S  C  I  Y  S  A  L  S  N  T
 901 tccccagttaacatgcaagtgctggctctcccaccaccccttcctaagacgcagcctggg
 301  S  P  V  N  M  Q  V  L  A  L  P  P  P  L  P  K  T  Q  P  G
 961 cccctctctctgaaacttcagattgccaaggataaaagctatggttcttactatggttct
 321  P  L  S  L  K  L  Q  I  A  K  D  K  S  Y  G  S  Y  Y  G  S
```

```
1021 gatgcctacccactggtaaaattcctccaggatcctatttatgtggaggtctccatcatt
 341  D  A  Y  P  L  V  K  F  L  Q  D  P  I  Y  V  E  V  S  I  I
1081 cacaggacagacccctcattgggtctgctgctagagcaatgttgggccacacctggctct
 361  H  R  T  D  P  S  L  G  L  L  L  E  Q  C  W  A  T  P  G  S
1141 aatccttttcatcaaccacagtggccaatcctggtgaagggatgcccatatgctggagac
 381  N  P  F  H  Q  P  Q  W  P  I  L  V  K  G  C  P  Y  A  G  D
1201 aactatcagaccaaaaggatccctgtccagaaagcatcaagtccctttccatctcatcac
 401  N  Y  Q  T  K  R  I  P  V  Q  K  A  S  S  P  F  P  S  H  H
1261 cagcgcttcagcatcgctaccttcagcttcatgagtgctgcaagggagaagcaggtttta
 421  Q  R  F  S  I  A  T  F  S  F  M  S  A  A  R  E  K  Q  V  L
1321 agtggacaggtatacctgcactgcagtgcatcagtctgccagcctgctggaatgccttcc
 441  S  G  Q  V  Y  L  H  C  S  A  S  V  C  Q  P  A  G  M  P  S
1381 tgtgtgatagtctgtcctgcttccaggagaagaagaaaatctgagctttattttgagaac
 461  C  V  I  V  C  P  A  S  R  R  R  R  K  S  E  L  Y  F  E  N
1441 acaaccagcatatctagcaaaggccctgtgatcctcctccaagccactaaggactctgca
 481  T  T  S  I  S  S  K  G  P  V  I  L  L  Q  A  T  K  D  S  A
1501 aacgtgcttcctagacactcaagcgcccctgtggattctcctgctctgtgggtaatgggg
 501  N  V  L  P  R  H  S  S  A  P  V  D  S  P  A  L  W  V  M  G
1561 ctttctgcaaccatgatcatcattggagtcttggtagtatcctacttggccatcagaaaa
 521  L  S  A  T  M  I  I  I  G  V  L  V  V  S  Y  L  A  I  R  K
1621 ttgagatga
 541  L  R  -
```
