# Supplementary material for: ZP4 Is Present in Murine Zona Pellucida and Is Not Responsible for the Specific Gamete Interaction
Source: Front Cell Dev Biol. 2021 Jan 18;8:626679. doi: 10.3389/fcell.2020.626679 (PMC7848090; doi:10.3389/fcell.2020.626679)
Supplement: Supplementary file 5 [file Image_3.pdf]

Fig. S3. Comparison of ZP1 amino acid sequences from *Homo sapiens*, *Mesocricetus auratus*, *Rattus norvegicus*, *Mastomys coucha*, *Mus pahari*, *Mus musculus* and *Mus mattheyi*. The accession numbers of the sequences used are: *H. sapiens* (NP_997224), *M. auratus* (ABS86997.1), *R. norvegicus* (NP_445961), *M. coucha* (XP_031240590.1), *M. pahari* (AYN07270.1), *M. musculus* (NP_033606), and *M. mattheyi* (AYN07269.1). Identical amino acids are marked by an asterisk (*), colon (:) represents conserved residues and a period (.) represents semi-conserved residues. The signal peptide is marked in pink. The trefoil domain is shown in blue. The ZP module is shown in red. The consensus furin cleavage-site is underlined. The transmembrane domain is marked in orange. Cysteine residues are marked in green. The potential N-glycosylation sites are shown in purple.

```
H.sapiens         MAGGSATTWGYPVALLLLVA-TLGLGRWLQPDPGLPGLRHSYDCGIKGMQLLVFPRPGQT    59
M.auratus         ------MAWGCFVAVLLLVATPLRLGQHLHS---KPGLEYSYDCGVQGMQLLVIPRSNQT    51
R.norvegicus      ------MAWGCFVVLLLLVAAPLRLGQHLHL---KPGFQYSYDCGVQGMQLLVFPRPNQT    51
M.coucha          ------MAWGCFVVLLLLVAAPLRLGQRLPL---KPGFEYSYDCGVRGMQLLVFPRPNQT    51
M.pahari          ------MGWGCFVALLLLAAAPLRLAQHLHL---EPGLEYSYDCGVRGMQLLVFPRPNQT    51
M.musculus        ------MAWGCFVVLLLLAAAPLRLGQRLHL---EPGFEYSYDCGVRGMQLLVFPRPNQT    51
M.mattheyi        ------MAVGVVCVVLLLAAAPLRLGQRLHL---EPGFEYSYDCGVRGMQLLVFPRPNQT    51
                           *   .:***.*  * *.: *      **:.:*****::******:** .**

H.sapiens         LRFKVVDEFGNRFDVNNCSICYHWVTSRPQEPAVFSADYRGCHVLEK-DGRFHLRVFMEA    118
M.auratus         IRFKVLDEFGNRFEVNNCSICYHWVISEPHDPAVFSADYRGCHVLQK-DGRFHLRVFVQA    110
R.norvegicus      IQFKVLDEFGNRFEVNNCSICYHWVISEAQKPAVFSADYKGCHVLEKQDGRFHLRVFIQA    111
M.coucha          IQFKVLDEFGNRFEVNNCSICYHWVTSEAQEPAVFSADYKGCHVLEK-DGQFHLRVFIQA    110
M.pahari          IESKVLDEFGNRFEVNNCSICYHWVTSEAQKRTVFSADYQGCHVLEK-DGRFHLRVFIQA    110
M.musculus        VQFKVLDEFGNRFEVNNCSICYHWVTSEAQEHTVFSADYKGCHVLEK-DGRFHLRVFIQA    110
M.mattheyi        VQFKVLDEFGNRFEVNNCSICYHWVSSEAQERTVFSAEYKGCHVLEK-DGQFHLRVFIQA    110
                  :. **:*******:*********** *. :. :****:*:*****:* **:******::*

H.sapiens         VLPNGRVDVAQDATLICPKPDPSRTLDSQLAPPAMFSVSTPQTLSFLPTSGHTSQGSGHA    178
M.auratus         VLPNGYVDTAQDVTLICPKADHTVTPDPYLAPPTTPQPFTPHTFVPHTNSGHTLAGSGHT    170
R.norvegicus      VLPNGRVDTAQDVTLICPKPDHILTPESYLAPPTTPQPFIPHTFALHPISGHTLAGSGHT    171
M.coucha          VLPNGRVDTAQDVTLICPKPDRIVTRDPYLAPPTTPEPFTPHTFALHPITNHTLAGSGYS    170
M.pahari          VLPNGRVDIARDVTLICPKPDHIMTPDPYLAPPTTPEPFTPPTFALHSIPGHTLAGSGHT    170
M.musculus        VLPNGRVDIAQDVTLICPKPDHTVTPDPYLAPPTTPEPFTPHAFALHPIPDHTLAGSGHT    170
M.mattheyi        VLPNGRVDVAQDVTLICPKPDHIMTPDPYLAPPTTPEPFTPHTFALHLIPGHTLAGSGHT    170
                  ***** ** *:*.****** *   * :  ****:  .   * ::      .**  ***::

H.sapiens         FPS--------PLDPGH-SSVHPTPALPSPGPGPTLATLAQPHWGTLEHWDVNKRDYIGT    229
M.auratus         LAGSGHTPLLSTLYPEH-SFIHSTPAPPSPGPGPAGPTVPHPQWGTLEPLELTKLDSVGT    229
R.norvegicus      -------G-LTTLYPE----THPTPAPPSSEPGPVGPTVPQSQWGTLGSWELTELDSIGT    219
M.coucha          -------G-LTTLYPEHSSFTHPTPATPSPGPGLAGPTVPHPQWGTLEPWELTEMDSVGT    222
M.pahari          -------G-LTTLYSEH-SFTHPTPAPPSPGPGPAGPTVPHSQWGTLEPWELTELDSVGT    221
M.musculus        -------G-LTTLYPEQ-SFIHPTPAPPSLGPGPAGSTVPHSQWGTLEPWELTELDSVGT    221
M.mattheyi        -------G-LTTLYPEH-SFTHPTPAPPSPGPGPAGPTAPHSQWGTLEPWELTELDSVGT    221
                              *        * *** **  ** .  *  : :****   ::.: * :**

H.sapiens         HLSQEQCQVASGHLPCIVRRTSKEACQQAGCCYDNTREVPCYYGNTATVQCFRDGYFVLV    289
M.auratus         HLTQEQCQVASGHIPCMIKSSSKEACQQAGCCYDNTREVPCYYGNTATLQCSRSGYFTLA    289
R.norvegicus      HLLQERCQVASGHIPCMVKGSSEEACQQAGCCYDNTKEMPCYYGNTVTLQCFRSGYFTLV    279
M.coucha          HLPQERCRVASGHIPCMVKGSSKEACQQAGCCYDNTKEEPCYYGNTVTLQCFKSGYFTLV    282
M.pahari          HLPQEQCQVASGHIPCMVKGSSKEACQQAGCCYDSTKEEPCYYGNTVTLQCFKSGYFTLV    281
M.musculus        HLPQERCQVASGHIPCMVNGSSKETCQQAGCCYDSTKEEPCYYGNTVTLQCFKSGYFTLV    281
M.mattheyi        HLPQEQCQVSSRHIPCMVKGSPKEACQQAGCCYDSAKEEPCYYGNTVTLQCFKSGYFTLV    281
                  ** **:*:*:* *:**::. : :*:*********.::* *******.*:** :.***.*.

H.sapiens         VSQEMALTHRITLANIHLAYAPTSCSPTQHTEAFVVFYFPLTHCGTTMQVAGDQLIYENW    349
M.auratus         ISQETALTHRVMLNNIHLAYAPSRCPPTQKTSAFVVFHVPLTLCGTTIQVVGEQLIYENQ    349
R.norvegicus      MSQETALTHGVMLDNVHLAYAPNGCPPTQKTSAFVVFHVPLTLCGTAIQVVGKQLVYENQ    339
M.coucha          MSQEMALTHGVMLDNVHLAYAPNGCPPTQKTSAFVVFHVPLTLCGTAIQVVGEQLIYENQ    342
M.pahari          VSQETALTHGVMLDNVRLAYAPNGCPPTQKTSAFVVFHVPLTLCGTAIQVVGEQLIYENQ    341
M.musculus        MSQETALTHGVLLDNVHLAYAPNGCPPTQKTSAFVVFHVPLTLCGTAIQVVGEQLIYENQ    341
M.mattheyi        MSQETALTHGVMLDNVHLAYASNGCPPTQKTSAFVVFRVPFTLCGTTIQVVGEQLIYENQ    341
                  :*** **** : * *::**** . * ***:*.***** .*:* ***::**.*.**:***
```

```
H.sapiens          LVSGIHIQKGPQGSITRDSTFQLHVRCVFNASDFLPIQASIFPPPSPAPMTQPGPLRLEL    409
M.auratus          LVSNIDVQKGPKGSITRDSVFRLHVRCIFNASDFLPVQASIFSPQPPAPVTQSGPLRLEL    409
R.norvegicus       LVSNIEVQTGPQGSITRDGVFRLHVRCIFNASDFLPIRASIFSPQPPAPVTRSGPLRLEL    399
M.coucha           IVSDMDVQEGLQGSITRDSVLRLHVRCIFNASDFLPIQASIFSPQPPSPVIQSGPLRLEL    402
M.pahari           LVSDIDIQKGPQGSITRDSVFRLHVRCIFNASDFLPIQASILSPQPPAPVTQSGPLKLEL    401
M.musculus         LVSDIDVQKGPQGSITRDSAFRLHVRCIFNASDFLPIQASIFSPQPPAPVTQSGPLRLEL    401
M.mattheyi         LVSDIDVQKGPQGSITRDSVFRLHVRCIFNASDFLPIQASIFSPQPPAPVTQSGPLRLEL    401
                   :**.:.:* * :******..::*****:********::***: *  *:*: : ***:***

H.sapiens          RIAKDETFSSYYGEDDYPIVRLLREPVHVEVRLLQRTDPNLVLLLHQCWGAPSANPFQQP    469
M.auratus          RIAKDKTFSSYYRERDYPLARLLQEPVHVEIRLLQRTDPGMVLMLHQCWATPTANPFQQP    469
R.norvegicus       RIATDKTFSSYYQGSDYPLVRLLQEPVYIEVRLLQRTDPGLALMLHQCWATPSASPFEQP    459
M.coucha           RIATDKTFSSYYQGSDYPLVRLLQEPVYVEVRLLQRTDPSLVLVLHQCWATPTTNPFEQP    462
M.pahari           RIATDKTFSSYYQGSDYPLVRLLQEPVYIEVRLLQRTDPSLVLVLHQCWATPTTSPFEQP    461
M.musculus         RIATDKTFSSYYQGSDYPLVRLLREPVYVEVRLLQRTDPSLVLVLHQCWATPTTSPFEQP    461
M.mattheyi         RIATDKTFSSYYQGSDYPLVRLLQEPVYVEVRLLQRTDPSLVLVLHQCWATPTASPIEQP    461
                   ***.*:******   ***:.***:***::*:********.:.*:*****.:*::.*::**

H.sapiens          QWPILSDGCPFKGDSYRTQMVALDG-ATPFQSHYQRFTVATFALLDSGSQRALRGLVYLF    528
M.auratus          QWPILSDGCPFEGDNYRTQMVALDRAELLFWSHYRRFTVTTFTLLDSSAGSTLRGLVYFF    529
R.norvegicus       QWPILSDGCPFKGDNYRTQMVAADRATLPFWSHYQRFTIATFTLLDSSSQNALRGQVYFF    519
M.coucha           QWPILSDGCPFKGDNYRTQLVAADKEALSFWSHYRRFTIATFTLLDSSSQNALRGQVYFF    522
M.pahari           QWPILSDGCPFKGDNYRTQVVAADREALPFWSHYQRFTIATFTLLDSSSQNALRGQVYFF    521
M.musculus         QWPILSDGCPFKGDNYRTQVVAADREALPFWSHYQRFTITTFMLLDSSSQNALRGQVYFF    521
M.mattheyi         QWPILSDGCPFKGDNYRTQVVATDKEALPFWSHYQRFTIATFTLLDFSSQNALRGQVYFF    521
                   ***********:**.****:** *     * ***:***::** *** .:  :*** **:*

H.sapiens          CSTSACHTSGLETCSTACSTGTTRQRRSSGHRNDTARPQDIVSSPGPVGFEDSYGQEPTL    588
M.auratus          CSASVCYPEGSETCSTVCDSGMARHRRSTGHHNSTVHALDIVSSPGAVGFEDAAK----L    585
R.norvegicus       CSASACHPVGSETCSTTCDSEIARHRRSSGHHNSTIRALDIVSSPGAVGFEDAPK----L    575
M.coucha           CSASACHPVGSKTCSTTCDSGIARRRRSSGHHNSTIQALNIVSSPGAVGFEDAAK----L    578
M.pahari           CSASACHPVGSNTCSTTCDSGIARRRRSSSHHNSVLRALDIVSSPGAVGFEDAAK----L    577
M.musculus         CSASACHPLGSDTCSTTCDSGIARRRRSSGHHNITLRALDIVSSPGAVGFEDAAK----L    577
M.mattheyi         CSASACHPVGSDTCSTTCDSGIARRRRSSGHHNSTLRALDIVSSPGAVGFEDTAK----L    577
                   **:*.*:  * .****.*.:  :*:***:.*:* . :  :****** *****:      *

H.sapiens          GPTDSNGNSSLRPLLWAVLLLPAVALVLGFGVFVGLSQTWAQKLWESNRQ    638
M.auratus          KPSGSSRNSISRPLLWVLLLL-LVTTLVLMSL------------------    616
R.norvegicus       EPSGSTRNSGSRPLLWVLQLL-ALTLVLGDGVLVGLSWAWAWA-------    617
M.coucha           EPSGSSRNSSSRMLL---LLL-AITLALGAGIFVGLIWAWAQKLWESIRH    624
M.pahari           EPSGSSRSSSSRVLL---PLL-AVTLAMAAGIFVGLIWAWAQKLWEGIRY    623
M.musculus         EPSGSSRNSSSRMLL---LLL-AITLALAAGIFVGLIWAWAQKLWEGIRY    623
M.mattheyi         EPSGSSRNSSSRMLL---LLL-AITLALAAGIFVGLIWAWAQKLWEGIRY    623
                    *:.*. .*  * **    **  ::  :  .:
```
