# Supplementary material for: ZP4 Is Present in Murine Zona Pellucida and Is Not Responsible for the Specific Gamete Interaction
Source: Front Cell Dev Biol. 2021 Jan 18;8:626679. doi: 10.3389/fcell.2020.626679 (PMC7848090; doi:10.3389/fcell.2020.626679)
Supplement: Supplementary file 6 [file Image_4.pdf]

Fig. S4. Comparison of ZP4 amino acid sequences from *Homo sapiens*, *Mesocricetus auratus, Rattus norvegicus*, *Mastomys coucha, Mus pahari* and *Mus mattheyi*. The accession numbers of the sequences used are: *H. sapiens* (NP_067009), *M. auratus* (ABH06548.1), *R. norvegicus* (NP_758833), *M. coucha* (XP_031215126), *M. pahari* (AYN07268) and *M. mattheyi* (AYN07267). Identical amino acids are marked by an asterisk (*), colon (:) represents conserved residues and a period (.) represents semi-conserved residues. The signal peptide is marked in pink. The trefoil domain is shown in blue. The ZP module is shown in red. The consensus furin cleavage-site is underlined. The transmembrane domain is marked in orange. Cysteine residues are marked in green. The potential N-glycosylation sites are shown in purple.

```
H.sapiens        ---------MWLLRCVLLCVSLSLAVSGQHKPEAPDYSSVLHCGPWSFQFAVNLNQEATS   51
M.auratus        MASRTLSSTLWLLPGIFLCFPFCPPLSGQHVTELP---GVLHCGLGSFQFTVNLSLEAES   57
R.norvegicus     MARQALRSTLWLLPSILLCFPFCLPLSGQHVTELP---GVLHCGLQSFQFAVNLSLEAES   57
M.coucha         MARQALRSTPWLLLSVLLCFPFCPPLSGQHVTELS---GVLRCRLQDFQFTVNLSLETEN   57
M.pahari         MAGQALRSTLWLLPSIFLCFPFCPPLSGQHVTELP---GVLHCRLQSFQFTVNLSLEAES   57
M.mattheyi       MAGQALRSTLWLLPSILLCFPFCPPLSGQHVTELP---GVLHCGLQSFQFTANLSLGAES   57
                           ***  ::**. :.  :****  *     .**:*   .***:.**.  : .

H.sapiens        PPVLIAWDNQGLLHELQNDSDCGTWIRKGPGSSVVLEATYSSCYVTEWDSHYIMPVGVEG   111
M.auratus        -PVLTAWDSRGLPHRLKNDSDCGTWVMDSPGDSLVLEATYNGCYVTMSSSHYVMEVGVQD   116
R.norvegicus     -PVLTTWDSQGLPHRLKNDSDCGTWVMDSPDGFLVLEASYSGCYVTLEGSHYIMTVGVQE   116
M.coucha         -PVLTAWDSQGLPHRLKNDSDCGTWVIDSPDGFLVLEATYTGCYVTLEGSHYVMMVGMQE   116
M.pahari         -PVLTAWDSQGLPHRLKNDSDCGTWVMDSPDGFLVLEATYNGCYVTLEGSHYVMMVGVQE   116
M.mattheyi       -PVLTAWDSQGLPHRLKNDSDCGIWMTDSPDGFLVLKATYNGCYVTLKGSHYVMMVSMQE   116
                  *** :**.:** *.*:****** *: ..*.. :**:*:*..****  .***:* *.::

H.sapiens        AGAAEHKVVTERKLLKCPMDLLA--RDAPDTDWCDSIPARDRLPCAPSPISRGDCEGLGC   169
M.auratus        VNVTEHMPGARKRLLKCPLDRQG--PNTLSTEVCNPVPVKERLLCAPLPISQGDCDKLGC   174
R.norvegicus     ADVAGHVAGTRQRLLTCPLALQGKAPDTPSAKVCSPVPVKERLPCASSTISRGDCEELGC   176
M.coucha         VDVAGNMTRTRERLLKCPLDLPSKAPDAPSAEVCSPVPIKERLPCAPSPISRGDCEEVGC   176
M.pahari         VDVAGNITGRRERLLKCPVDLHT--TDASNAEVCSPVPVKERLPCAPSPISRGDCEEAGC   174
M.mattheyi       VDVAGNMTGTRERLLKCPLDLRA--PDAPSAEVCSPVPVKERLPCAPSPISRGDCEEVGC   174
                 ...: :    ..:**.**:       :: .:. *. :* ::** **   **:***:  **

H.sapiens        CYSSE--EVNSCYYGNTVTLHCTREGHFSIAVSRNVTSPPLLLDSVRLALRN-DSACNPV   226
M.auratus        CYIAEEEEVGYCYYGNTVTSQCSREGSFSIAVSRNVTSPPLNLDSLHLVVRS-DSGCDPV   233
R.norvegicus     CYSSEEEGADSCYYGNTVTSHCTKEGHFSIAVSRDVTSPPLRLDSLRLGFRNITTGCDPV   236
M.coucha         CYSSEEEEAGSCYYGNTVTSHCTREGGFSIVVSRNATSPPLRLDSLRLVSRN-NSGCDPV   235
M.pahari         CYSSEEEEAGSCYYGNTVTSRCTREGRFSIAVSRNATSPPLHLDSLRLVFRD-NSACDPV   233
M.mattheyi       CYSSEEEEAGSCYYGNTVTSRCTREGRFSIAVSRNATSPPLRLDSLHLVFRN-SSGCDPV   233
                 ** :*   .. ******** :*::** ***.***:.***** ***::*  *.  :.*:**

H.sapiens        MATQAFVLFQFPFTSCGTTRQITGDRAVYENELVATRDVKNGSRGSVTRDSIFRLHVSCS   286
M.auratus        MATPTFALFQFPFTSCGTTRRVIGDQVVYENELLATQDVRTWGNGSITRDSIFRLRVSCS   293
R.norvegicus     MKTSTFVLFQFPLTSCGTTQRITGDQAMYENELVAIRDVQAWGRSSITRDSNFRLRVSCT   296
M.coucha         MTTSTFVLFQFPFSSCGTTRRITGDQALYENELVAIQDVQAWGRSSITRDSNFRLRVSCT   295
M.pahari         MTTATFVLFQFPFTSCGTTRRITGDKAVYENELVAIRDVQAWGRSSITRDSNFRLRVSCI   293
M.mattheyi       MMTSTFVLFQFPFTSCGTARRITGDQAVYENELVAIPDVQAWGRSSITRDSNFRLRVSCT   293
                 * * :*.*****::****:::: **:.:*****:*  **:  ...*:**** ***:***

H.sapiens        YSVSSNSLPINVQVFTLPPPFPETQPGPLTLELQIAKDKNYGSYYGVGDYPVVKLLRDPI   346
M.auratus        YSVLSNTSPINMQVLTLPPPLPKTQPGSLSLELQIAKDETYGSYYGAEDYPLVKFLQDPI   353
R.norvegicus     YSIHSIMSPVNMQVWTLPPPLPKTQPGPLSLELQIAQDKNYSSYYGTDAYPLVKFLQDPI   356
M.coucha         YSVHSNTSPVNIRVLALPPPIPKTQPGPLSLELQIAKDKSYGSYYGSDAYPLVKFLQDPI   355
M.pahari         YSALSNTSPVNMQVLALPPPLPKTQPGPLSLKLQIAKDKSYGSYYGSDAYPLVKFLQDPI   353
M.mattheyi       YSALSNTSPINMQVLALPPPLPKTQPGPLSLELQIAKDKSYGSYYGSDAYPLVKFLQDPI   353
                 **  *   *:*::* :****:*:**** *:*:****:*:.*.****   **:**:*:***

H.sapiens        YVEVSILHRTDPYLGLLLQQCWATPSTDPLSQPQWPILVKGCPYIGDNYQTQLIPVQKAL   406
M.auratus        YVEVSILHRTDPSLELLLEQCWATSGPNPFLQPQWPILVKGCPYAGDNYQTRRINVQKAS   413
R.norvegicus     YVEVSILHRTDPSLSLLLEQCWATPGSNPFHQPQWPILVKGCPYAGDNYQTKRIPVQKAS   416
M.coucha         YVEVSILHRTDPSLGLRLEQCWATPGSNPFHQPQWPILVKGCPYAGDNYQTKRIPVQKAS   415
M.pahari         YVEVSIIHRTDPSLGLLLEQCWATPGSNPFHQPQWPILVKGCPYAGDNYQTKRIPVQKAS   413
M.mattheyi       YVEVSIIHRTDPSLGLLLDQCWATPGSNPFHQPQWPILVKGCPYAGDNYQTKRIPVQKTS   413
                 ******:***** * *:***** . :*: ************  ******: * ***:
```

```
H.sapiens         DLPFPSHHQRFSIFTFSFVNPTVEKQALRGPVHLHCSVSVCQPAETPSCVVTCPDLSRRR    466
M.auratus         -RPFPSHHQRFSISTFSFTNAIRKGQSFAGQVYLHCSALVCQPAGTPSCKAICPASRRRR    472
R.norvegicus      -DVFPSHHQRFSISTFSFMSAGREKQVLGGQVYLHCSASVCQPAGMPSCTVICPASRRRR    475
M.coucha          -GPFPSHRQRFSIATFSFMSAVREKQVLGGQVYLHCSASVCQPAGMPSCMIICPASRRRR    474
M.pahari          -SPFPSHHQRFSIATFSFMSAAREKQVLSGQVYLHCSASVCQPAGMPSCVIVCPASRRRR    472
M.mattheyi        -SPFPSHHQRFSIDTFSFMSAVREKQLLSGQVYLHCSASVCQPAGMPSCVTVCPASRRRR    472
                     ****:***** **** .   : * : * *:****. *****  ***   **   ***

H.sapiens         NFDNSSQNTTASVSSKGPMILLQATKDPPEKL----RVPVDSKVLWVAGLSGTL-ILGAL    521
M.auratus         KSELYFKNNTARISSKGPVILLQATKDPADMLHRYSSTPMNSPALWVVGLSAITIIISIL    532
R.norvegicus      KSELYFDNS-TSISSKGPVILLQATKDPAVMLHKHSGTHADSPTLWVMGLSASMVITGVL    534
M.coucha          KSELYFENT-TSVSSKGPVILLQATKDPAETLHRYSSTPVDSPALWVVGLSATVIVIGVL    533
M.pahari          KSELYFENT-TSISSKGPVILLQATKDSANVLPRHSSAPVDSPALWVMGLSATMIIIGVL    531
M.mattheyi        KSELHFETT-TSISSKGPLILLQATKDSADMLHRHSRTPVDSTALWVMGLSATVIITGVF    531
                  : :   ... : :*****:********    *     .  :* .*** ***.   : . :

H.sapiens         LVSYLAVKKQKSCPDQMCQ    540
M.auratus         LVFYLAIRKAR--------    543
R.norvegicus      VVSYLATRKQR--------    545
M.coucha          VGSYLAIRKWR--------    544
M.pahari          VVSYLAIRKLR--------    542
M.mattheyi        VVSYLAIRKLR--------    542
                  :  *** :* :
```
